# Skull morphological evolution in Malagasy endemic Nesomyinae rodents

**DOI:** 10.1371/journal.pone.0263045

**Published:** 2022-02-04

**Authors:** Léa Terray, Christiane Denys, Steven M. Goodman, Voahangy Soarimalala, Aude Lalis, Raphaël Cornette

**Affiliations:** 1 Institut de Systématique, Evolution, Biodiversité (ISYEB), Muséum national d’Histoire naturelle, CNRS, SU, EPHE, UA, CP 51, Paris, France; 2 Field Museum of Natural History, Chicago, IL, United States of America; 3 Association Vahatra, Antananarivo, Madagascar; 4 Institut des Sciences et Techniques de l’Environnement, University of Fianarantsoa, Fianarantsoa, Madagascar; Liverpool John Moores University, UNITED KINGDOM

## Abstract

Madagascar is a large island to the south-east of Africa and in many ways continental in size and ecological complexity. Here we aim to define how skull morphology of an endemic and monophyletic clade of rodents (sub-family Nesomyinae), that show considerable morphological variation, have evolved and how their disparity is characterized in context of the geographical and ecological complexity of the island. We performed a two-dimensional geometric morphometric analysis on 370 dorsal and 399 ventral skull images of 19 species (comprising all nine extant endemic genera) and tested the influence of three ecological parameters (climate, locomotor habitat and nychthemeral cycle) in a phylogenetic context on size and shape. The results indicate that skull shape appears to importantly reflect phylogeny, whereas skull size does not carry a significant phylogenetic signal. Skull shape is significantly influenced by climate while, skull size is not impacted by any of the ecological factors tested, which is controversial to expectations in an insular context. In conclusion, Nesomyinae must have evolved under unusual types of local constraints, preventing this radiation from demonstrating strong ecological release.

## Introduction

Madagascar is a large island [1] (nearly 590,000 km^2^) situated about 400 km off the southeastern coast of Africa. It is the 4^th^ largest island on the planet and aspects of its biogeography are unique among other large islands in the tropics. Indeed, Madagascar has a large surface area associated with geological and meteorological complexities: highly different environments co-exist on this island as rainforests, steppes or karstic deserts. Madagascar has greater ecosystem richness than any other island [2, 3]. This fact is supported by the high rate of endemism observed at different taxonomic levels, resulting in this island being considered as a biodiversity hotspot [4, 5]. This diversity is illustrated by the four extant groups of endemic living Malagasy land mammals (Lemuroidea, Eupleridae, Tenrecidae and Nesomyinae), representing several hundred of species [6, 7].

Each endemic mammal clade is the result of an independent successful colonization event. Nesomyinae colonized Madagascar in the early Miocene and probably originated from eastern Africa [6, 8, 9]. This monophyletic group is divided into two main clades and is currently (as of late 2018) composed of nine genera and 30 recognized extant species, all living in the diverse native forest ecosystems of the island [10]. Because this sub-family is endemic to Madagascar, it represents a unique opportunity to characterize, at macroevolutionary level, its radiation and estimate the importance of ecology, considered in a phylogenetic context, in shaping morphological diversity. The skull is an ideal structure for this type of investigation. Because it carries structures related to sensory functions (vision, olfaction, taste, etc.), feeding, and locomotion [11–13], it is likely to be influenced by environmental factors [12, 14–16].

In this paper, we addressed the following question: in this particular geographical and ecological context of Madagascar, what shaped the morphological diversity observed in extant Nesomyinae rodents? To better understand the patterns and processes of evolution of the Nesomyinae, we examined the two following sub-questions: 1) To what extent does the skull shape of Nesomyinae reflect their phylogenetic history? 2) Did environmental parameters significantly influenced the shape of the skull and if so, how? To answer those questions, we performed shape analysis of Nesomyinae skulls in dorsal and ventral views, using geometric morphometrics (here abbreviated as GM). Then, we assessed the significance of phylogenetic signal and tested the influence of ecology on the skull shape and size. We expect that the skulls of different nesomyines, and especially size, to display adaptations to local environments, as insular context is known to favor rapid character displacement towards local optima [17]. However, Madagascar being a particular case with several geographical and ecological continental characteristics more at a continental level, typical insular evolutionary trends [17] might not be observed. In addition, skull morphology can also show low evolutionary lability because of the strong phylogenetic signal in teeth, that are morphologically conserved [11, 18]. In this case, because of the strength of phylogenetic signal, we would expect Nesomyinae skull to be less influenced by ecological variability.

## Materials and methods

The protocols employed to treat these data provided here are available on line at:

Denys, Christiane; Terray, Lea; Cornette, Raphael (2021), “Nesomyinae (Rodentia,Mammalia) protocoles for skull form evolution study”, Mendeley Data, V1, https://data.mendeley.com/datasets/65828588fv/1 (doi: 10.17632/65828588fv.1)

The original TPS data and nexus file for the phylogenetic tree have been stored in OSF site:

https://osf.io/a8eth/?view_only=abd1896945ee4bbd83cbbbead4c736ea

### Sampling

We used a data set of Nesomyinae skull photographs taken with a macro-photographic CANON EOS including 370 dorsal and 399 ventral images. The images were collected in a standardized way to prevent any bias due to the effect of parallax [19]: in dorsal view the frontal part of the skull was horizontally oriented (parallel to the photographic plane), and in ventral view molar rows were oriented as to be parallel to the photographic plane. Juveniles (defined as having portions of the skull being unossified) and older individuals (with heavily worn teeth) are not included in our sample. To minimize any potential bias due to sexual dimorphism we have included for each species as many specimens as possible and of both sexes; although, we add that this subfamily is not known to show sexual dimorphism [20]. Several species are known by only one or few individuals, such as *Brachytarsomys villosa*. The list of specimens used herein is presented in **S1 Table**. These are housed in the Field Museum of Natural History (FMNH), Chicago; The Natural History Museum (formerly British Museum of Natural History [BMNH]), London; the Mention Zoologie et Biodiversité Animale (formerly Département de Biologie Animale), Université d’Antananarivo (UADBA), Antananarivo, Madagascar; the Museum für Naturkunde (ZMB), Berlin; and the Muséum national d’Histoire naturelle (MNHN), Paris. A summary of the specimens is presented in **Table 1**.

**Table 1:** Summary of the photographic sampling.

	DORSAL VIEW			VENTRAL VIEW		
Species	Number of individuals	Sex	Inclusion of referent specimen (Yes/No)	Number of individuals	Sex	Inclusion of referent specimen (Yes/No)
*Eliurus carletoni*	28	F(14) M(13)	Yes	30	F(19) M(10)	Yes
*Eliurus majori*	29	F(14) M(13)	Yes	32	F(14) M(15)	Yes
*Eliurus antsingy*	5	F(2) M(3)	No	5	F(2) M(3)	No
*Eliurus grandidieri*	30	F(11) M(18)	Yes	33	F(13) M(19)	Yes
*Eliurus minor*	25	F(11) M(12)	Yes	26	F(11) M(13)	Yes
*Eliurus myoxinus*	25	F(13) M(10)	No	26	F(13) M(11)	No
*Eliurus tanala*	30	F(16) M(11)	Yes	31	F(15) M(13)	Yes
*Eliurus webbi*	22	F(7) M(14)	Yes	23	F(8) M(14)	Yes
*Voalavo gymnocaudus*	10	F(4) M(4)	Yes	11	F(4) M(4)	Yes
*Gymnuromys roberti*	33	F(21) M(10)	Yes	36	F(22) M(11)	Yes
*Brachytarsomys albicauda*	9	F(4) M(2)	Yes	9	F(4) M(2)	Yes
*Brachytarsomys villosa*	3	F(0) M(3)	Yes	3	F(0) M(3)	Yes
*Brachyuromys betsileoensis*	19	F(10) M(6)	Yes	21	F(11) M(7)	Yes
*Brachyuromys ramirohitra*	11	F(1) M(7)	Yes	13	F(3) M(7)	Yes
*Nesomys audeberti*	12	F(4) M(5)	No	11	F(3) M(5)	No
*Nesomys rufus*	31	F(18) M(11)	Yes	34	F(19) M(13)	No
*Hypogeomys antimena*	15	F(3) M(4)	Yes	11	F(3) M(3)	Yes
*Monticolomys koopmani*	14	F(7) M(6)	No	24	F(14) M(9)	No
*Macrotarsomys bastardi*	19	F(8) M(6)	Yes	20	F(7) M(8)	Yes
**TOTAL**	370	-	-	399	-	-

M: Male, F: female. Reference specimen: holotype or paratype specimen.

### Morphometric analyses

**Fig 1:**
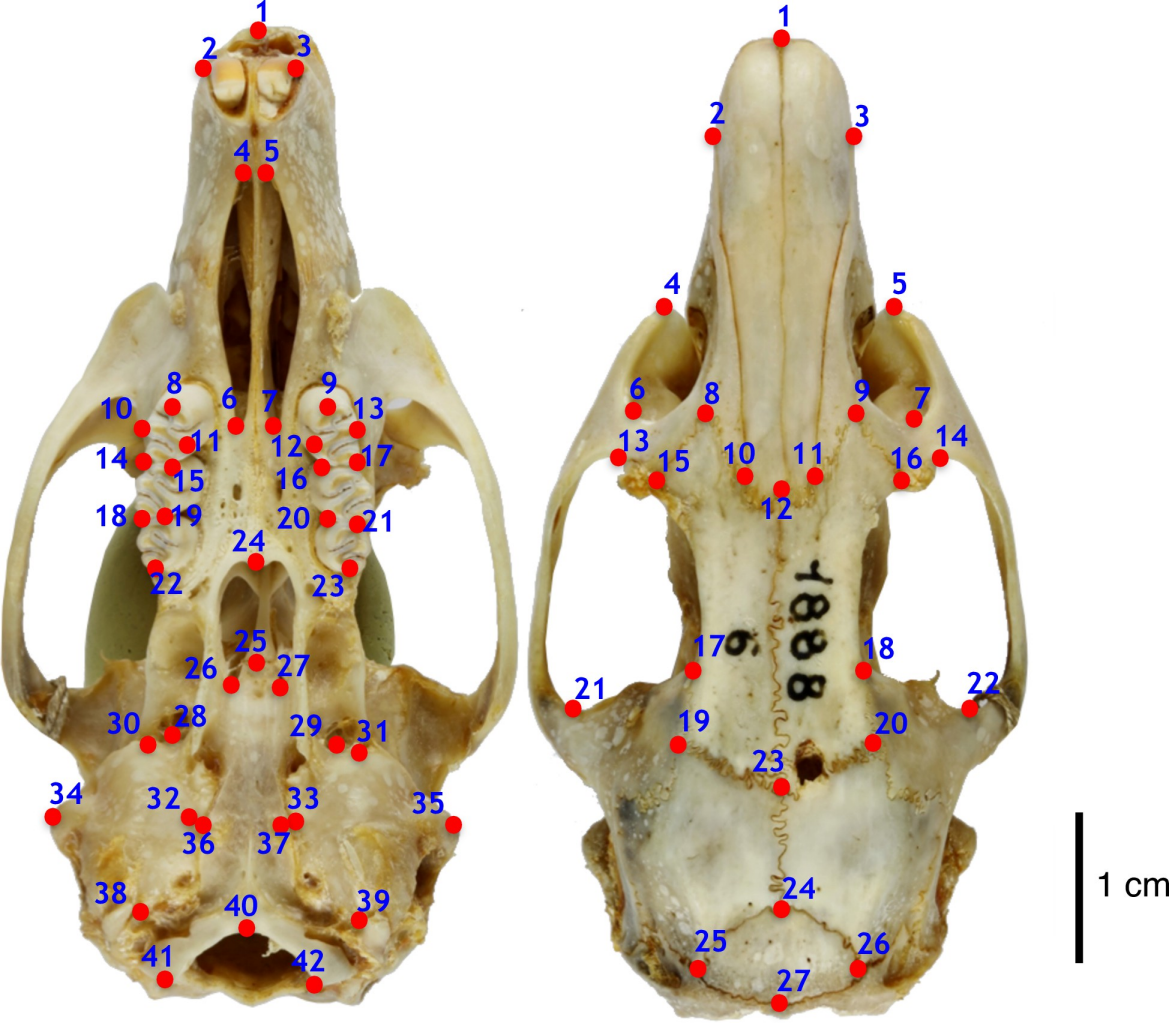
Landmarks locations presented on a skull of *Hypogeomys antimena* (MNHN ZM- 1888–6). Ventral view: 42 landmarks; dorsal view: 27 landmarks.

GM method allows a rigorous quantitative analysis of the geometric relationships of shape and size variation of an organism by combining a geometric concept of form with multivariate statistical procedures [21]. To capture skull shape variation, we used a 2-dimensional landmark-based approach. In dorsal view, 27 anatomical landmarks were chosen, as well as 42 in ventral view (**Fig 1**). Landmarks were selected to correspond as closely as possible to anatomical homologies. Descriptions of each type of landmark are given in **S2 and S3 Tables**. They have been digitized using the software tpsDig2 [22].

Ventral and dorsal data sets were analyzed separately. First, we performed a Generalized Procrustes Analysis (GPA). This method allows removal of effects due to scale, translation and rotation, maintaining only the geometric shape of objects and making comparisons possible [23]. This procedure was realized using the *gpagen* function of the *geomorph* library [24] under the free software R (R Core Team 2016).

We only examined the symmetric component of shape. Asymmetric component was explored using MorphoJ [25] and represents respectively 4.7% of shape variation in the ventral cranium and 5.8% in the dorsal cranium. It was removed using *bilat*.*symmetry* from *geomorph* [24]. Further statistical testing has also been performed under the free software R.

In order to reduce data dimensionality, principal component analyses (PCA) were performed on shape. PCA is a tool that uses the eigenvectors and eigenvalues of the covariance (or correlation) matrix to reduce the data dimensionality of a multivariate data set. The principal components are new uncorrelated variables (vectors loaded on the original variables) which successively maximize variance. This step was carried out using the *gm*.*prcomp* function of the *geomorph* library [24]. Visualizations of those PCA with all individuals are presented in **S1 Fig**. To reduce the number of variables we retained 95% of shape variation, the latest principal components being usually considered as negligible because they explain very little of the global shape variation [26]. Further analyses have been carried out on principal components instead of Procrustes coordinates.

Sexual dimorphism in ventral and dorsal view was tested when information was available (12 species: *Eliurus carletoni*, *Eliurus majori*, *Eliurus grandidieri*, *Eliurus tanala*, *Eliurus minor*, *Eliurus myoxinus*, *Eliurus webbi*, *Gymnuromys roberti*, *Brachyuromys betsileoensis*, *Nesomys rufus*, *Monticolomys koopmani* and *Macrotarsomys bastardi*). On shape we performed a Procrustes ANOVA with the function *procD*.*lm* from the package *geomorph* [24] using the formula: shape ~ sex + species + sex:species. For size we used the *lm* function from the *stats* package using the formula: size ~ sex + species + sex:species. In both cases, the “sex” term was examined to assess the presence of sexual dimorphism, and the interaction term to assess if sexual dimorphism is different between species.

Allometry is the part of shape due to the influence of size [27]. If the Procrustes superimposition method does separate size and shape, it does not remove allometry. Allometry at the interspecific level was investigated with *procD*.*lm* from *geomorph* [24] using the formula: shape ~ size + species + size:species. The log centroid size was used as an estimator of size. Interactions between species and size were examined to asses homogeneity of allometric slopes between species. This aspect was explored on all specimens of our sample in dorsal and ventral views.

All subsequent analyses have been performed on species means that include all specimens of a given taxa. For each data set comparative analyses were carried out on 1) shape, which correspond to the principal components computed on the symmetric component of Procrustes coordinates, and on 2) the centroid size, which is also obtained from the Procrustes superimposition method and is defined as the square root of the sum of square distance of each landmark from the centroid of the object.

### Phylogenetic signal

As a basis for phylogenetic analyses we used the phylogeny of muroid rodents of Steppan et al. [28], which is based on 900 muroid species. The tree was pruned to keep only species of interest using the function *keep*.*tip* of the library *ape* [29].

To quantify phylogenetic signal in size we used the K-statistic method for univariate traits [30]. To quantify it on shape we used the same method extended to multivariate data by Adams [31]. This approach compares observed traits variations to their expected variations under Brownian motion. If K-value = 1 the considered trait evolved according to Brownian motion. If tested groups resemble each other more than expected, i.e. strong phylogenetic signal, K-value >> 1. On the contrary, K-value close to 0 indicates no phylogenetic signal. This signal has been computed with *physignal* from *geomorph* [24].

To visualize to what extend shape reflect phylogeny, we performed PCA on mean shape per species and projected phylogeny on it. This step was performed using *phylomorphospace* from the *phytools* library [32]. Method for ancestral states reconstruction, morphometric branch lengths estimation and phylomorphospace reconstruction are described in Sidlauskas [33]. Visualization of shape variation along axes were obtained using *plotRefToTarget* from *geomorph* [24] and are deformations in comparison to the global mean shape.

### Influence of ecological factors

We tested the three best informed and relevant ecological parameters whose influence on mammalian skull morphology has been well documented: climate [34–36], locomotor habitat [12, 37–40] and nychthemeral cycle [41, 42]. Nesomyinae species occur in different natural vegetational zones of Madagascar, showing considerable local environmental variation [43] and, hence, these factors are good candidates to reflect adaptation. Based on recognized ecological characteristics of Nesomyinae [43], we assigned categories to characterize the three parameters: locomotor habitat (“terrestrial”, “arboreal”, and “semi-arboreal”), nychthemeral cycle (“nocturnal”, “twilight”, and “arrhythmic”) and climate (“tropical wet” and “hot and dry”) (**Table 2**). Specimens have been assigned to climatic areas based on the locality of their collection (**S1 Table**).

**Table 2:** Species and their associated ecological characteristics. Areas are generalized from collection localities, climate data are from the Direction Générale de la Météorologie de Madagascar [44] and data concerning locomotor habits and nychthemeral cycle are from Goodman and Soarimalala (2011) [43].

Species	Area(s)	Climate	Locomotorhabitat	Nycthemeral cycle
*Eliurus carletoni*	West coast	Tropical wet	Semi-arboreal	Nocturnal
*Eliurus majori*	East coast	Tropical wet	Arboreal	Nocturnal
*Eliurus antsingy*	West coast	Hot and dry	Terrestrial	Nocturnal
*Eliurus grandidieri*	East coast	Tropical wet	Terrestrial	Nocturnal
*Eliurus minor*	East coast	Tropical wet	Arboreal	Nocturnal
*Eliurus myoxinus*	East coast / West coast	Hot and dry	Arboreal	Nocturnal
*Eliurus tanala*	East coast	Tropical wet	Semi-arboreal	Nocturnal
*Eliurus webbi*	East coast	Tropical wet	Arboreal	Nocturnal
*Voalavo gymnocaudus*	East coast	Tropical wet	Arboreal	NA
*Gymnuromys roberti*	East coast / West coast / Central Highlands	NA	Terrestrial	Nocturnal
*Brachytarsomys albicauda*	East coast	Tropical wet	Arboreal	Nocturnal
*Brachytarsomys villosa*	East coast	Tropical wet	Arboreal	Nocturnal
*Brachyuromys betsileoensis*	East coast	Tropical wet	Terrestrial	Arrhythmic
*Brachyuromys ramirohitra*	East coast / Central Highlands	Tropical wet	Terrestrial	Arrhythmic
*Nesomys audeberti*	East coast	Tropical wet	Terrestrial	Twilight
*Nesomys rufus*	East coast / Central Highlands	Tropical wet	Terrestrial	Twilight
*Hypogeomys antimena*	West coast	Hot and dry	Terrestrial	Nocturnal
*Monticolomys koopmani*	East coast	Tropical wet	Terrestrial	NA
*Macrotarsomys bastardi*	West coast / South	Hot and dry	Terrestrial	Nocturnal

To quantify the influence of ecological factors on size, we performed ANOVA (F test), analyses of variance, which aims to determine whether qualitative factors (ecological factors) have significant effects on one quantitative variable (size). F is the ratio between inter- and intra-group variability. Thus, the more the average sizes of two groups are different, the higher the F statistic will be. Regarding shape we used MANOVA analyses (Wilks test). MANOVA, multivariate analysis of variances, is the extension of the ANOVA to multivariate data. It computes the λ of Wilks, which measures the part of intra-class inertia in total inertia. λ is comprised between 0 and 1, a value close to 0 indicating a good discrimination between the groups. When morphological descriptors found to carry significant phylogenetic signals we used phylogenetics MANOVA (MANOVA^phy^), which takes phylogeny into account for p-value estimation. We used *manova*.*gls* from *MvMORPH* [45]. Fit of generalized least square linear model was performed using penalized likelihood method which allows to better manage the biases due to the number of traits approaching the number of species [46]. Prior, four evolutionary models were tested and compared with the Generalized Information Criterion (GIC): Brownian Motion (BM) in which the quantity of evolutionary change in a trait is relative to branch length, Ornstein-Uhlenbeck (OU) which takes into account stabilizing/divergent selection and stasis implying that traits can evolve towards one or more optima, Early Burst (EB) that assumes an exponential reduction in diversification rates over time and Pagel’s lambda transformation (L) which scales the internal branches of the phylogeny thus reducing the expected covariance between species due to evolutionary history. To do that we used *GIC* from *MvMORPH* [45]. When no significant phylogenetic signal was found in morphological descriptors, the influence of ecological factors was determined using the function *aov* of the *Stats* library. For each case, ecological factors and their interaction with the log centroid size was tested using the formula: shape ~ size + ecology + size:ecology. Knowing that organisms reach different equilibrium sizes on islands as compared to continents, that is to say gigantism vs. dwarfism [17, 47], the interaction between ecological factors and size could provide additional insight into these patterns. For each model effect size was computed using the *effectsize* function from *MvMORPH* [45], which provide the estimator τ^2^ that take into account the penalized likelihood framework and can be interpreted relatively. The higher τ^2^, the stronger the association, and τ^2^<0 means no association. Because of missing data two species (*Voalavo gymnocaudus* and *Monticolomys koopmani*) were removed from nychthemeral cycle analyses. *Gymnuromys roberti* was removed from climate analyses as it is broadly distributed across different climatic zones.

When tests were significant, shape variations related to factors were investigated. We computed mean shape per category of each factor using *mshape* from *geomorph* [24].

## Results

### Morphometric analyses

No sexual dimorphism was detected in any of the species tested in dorsal size (sex: F = 2.86, p-value = 0.092; species: F = 55.37, p-value < 2e-16***; interaction: F = 1.57, p-values = 0.072), dorsal shape (sex: R^2^ = 0.0012, p-value = 0.15; species: R^2^ = 0.77, p-value = 0.001**, interaction: R^2^ = 0.013, p-value = 0.39), ventral size (sex: F = 0.008, p-value = 0.93; species: F = 42.24, p-value < 2e-16***; interaction: F = 0.77, p-value = 0.73), nor ventral shape (sex: R^2^ = 0.00071, p-value = 0.28; species: R^2^ = 0.8, p-value = 0.001**, interaction: R^2^ = 0.0089, p-value = 0.84).

The test of allometry was statistically significant in dorsal (size: R^2^ = 0.13, p-value = 0.001**; species: R^2^ = 0.65, p-value = .001**; interaction: R^2^ = 0.024, p-value = 0.001**) and ventral view (size: R^2^ = 0.051, p-value = 0.001**; species: R^2^ = 0.75, p-value = 0.001**; interaction: R^2^ = 0.024, p-value = 0.001**). Interaction between size and species is statistically significant in both cases, meaning that allometric slopes are heterogeneous between species. Plots of species allometric slopes are presented in **S2 Fig**.

### Phylogenetic signal

Results of K-statistics are presented in **Table 3**. In both data sets, centroid size has no statistically significant phylogenetic signal while a strong signal was detected in shape.

**Table 3:** Phylogenetic signal detected in size and shape.

	Data set	K value	P-value
**Shape**	Dorsal	0.90	0.001**
	Ventral	0.96	0.001**
**Centroid size**	Dorsal	0.50	0.097
	Ventral	0.53	0.064

Asterisks indicates level of significance (*<0.05, **<0.01, ***<0.001).

**Fig 2:**
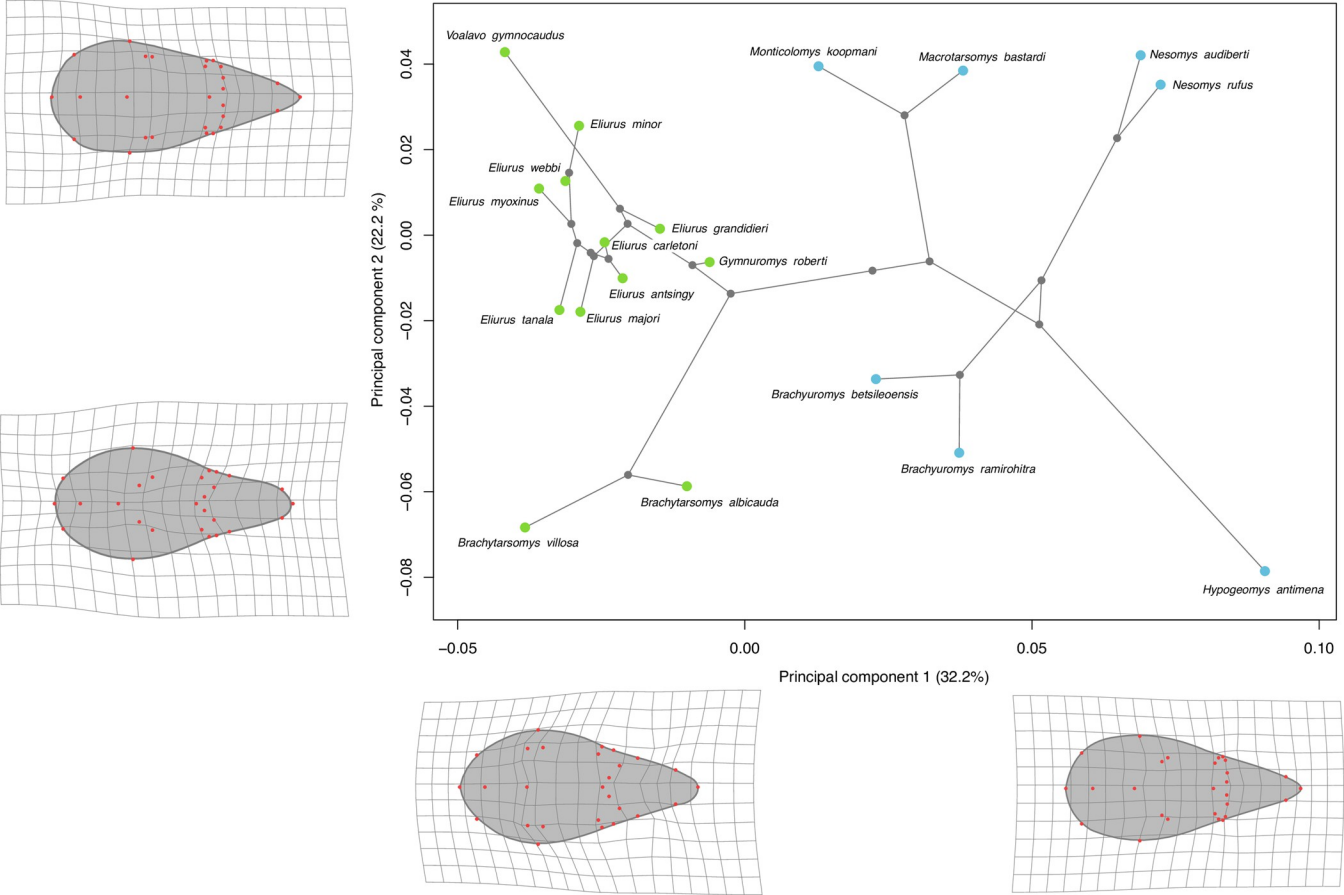
Visualization of the two first axes of the PCA performed on dorsal shape with phylogenetic projection. Colored points represent the morphological average of all individuals of a species. Colors indicate the two principal clades among Nesomyinae. Blue: clade formed by the genus *Brachyuromys*, *Nesomys*, *Macrotarsomys*, *Monticolomys*, and *Hypogeomys*; green: clade formed by the genus *Brachytarsomys*, *Eliurus*, *Gymnuromys*, and *Voalavo*. Warpgrids indicate shape variation along axis with maximum deformation observed at each extremity of the axis.

**Fig 3:**
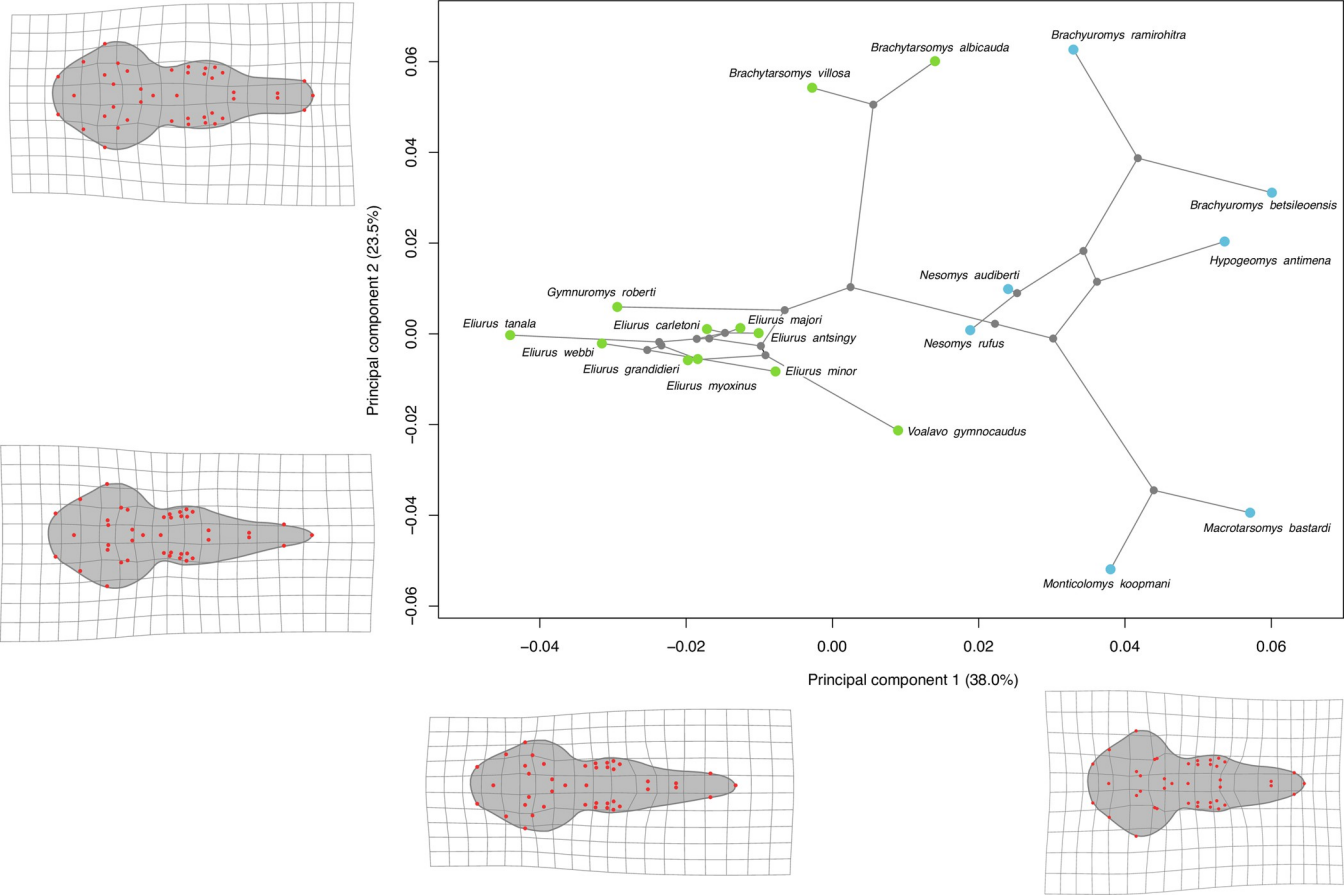
Visualization of the two first axes of the PCA performed on ventral shape with phylogenetic projection. Colored points represent the morphological average of all individuals of a species. Colors indicate the two principal clades among Nesomyinae. Blue: clade formed by the genus *Brachyuromys*, *Nesomys*, *Macrotarsomys*, *Monticolomys*, and *Hypogeomys*; green: clade formed by the genus *Brachytarsomys*, *Eliurus*, *Gymnuromys*, and *Voalavo*. Warpgrids indicate shape variation along axis with maximum deformation observed at each extremity of the axis.

In **Figs 2** and **3** the PCA with phylogenetic projection performed on respectively dorsal and ventral data sets are presented.

Regarding dorsal data set, the two first axes of the PCA in total encompass 54.4% of the variability (**Fig 2**). The first axis, which explains 32.3% of the total shape variation, display variation in relative widths of the anterior and posterior portions of the skull. For species situated in the negative area of the axis, the back of the skull is narrower, nasal bones longer (with no general elongation of the skull), and orbits in a more anterior position. In contrast, for species in the positive part of the axis the posterior portion of the skull is proportionally wider, nasal bones shorter and orbits in a more posterior position. This axis distinguishes the two Nesomyinae clades. The second axis of the PCA explains 22.2% of total shape variation. For species situated in the negative part of this axis, the posterior part of the skull is proportionally wider at the level of the jugal bone, braincase narrower and skull shorter in length. For the species situated in the positive part of the axis the braincase is wider and skull proportionally longer. This axis separates *Brachytarsomys villosa* and *Brachytarsomys*. *albicauda* and *Hypogeomys antimena* from the other species. The distribution of species represented by their morphological average in morphological space reflect their phylogenetic relationship, as expected given the high phylogenetic signal (**Table 3**). A PCA on the third and fourth axes, explaining respectively 13.3% and 7.6% of shape variation, is presented in **S3 Fig**.

Concerning the ventral data set the two first axes carry 61.5% of the total shape variation (**Fig 3**). The first axis explains 38.0% of the variability and the general patterns are consistent with the observed shape variation in dorsal view. For species situated in the positive part of this axis, the posterior portion of the skull is proportionally wider and rostrum large and rounded. In the negative part of this axis the posterior portion of the skull is proportionally narrower and rostrum narrow and pointed. This axis clearly separates the two Nesomyinae clades. The second axis of the PCA accounts for 23.5% of the total shape variation and the major shape variation concerns the relative length of the skull. For species situated in the negative part of the axis the tympanic bullae are proportionally larger, rostrum longer and more pointed, and incisor foramens longer as compared to species in the positive of the axis. The distribution of species in morphological space reflect their phylogenetic relationship, as expected given the high phylogenetic signal (**Table 3**). A PCA on the third and fourth axes, explaining respectively 7.9% and 7.2% of shape variation, is presented in **S4 Fig**.

### Influence of ecological factors

Results of ANOVA and MANOVA^phy^ performed on size and shape of both data sets are presented in **Table 4**.

**Table 4:** Tests of ecological factors on shape and size.

			Ecological factors		
	Data set		Climate	Locomotor habitat	Nychthemeral Cycle
**Shape** (MANOVA^phy^)	Dorsal	Ecological factor	Wλ = 0.48	Wλ = 0.4	Wλ = 0.10
			τ ^2^ = 0.12	τ ^2^ = -0.20	τ ^2^ = 0.35
			P = 0.34	P = 0.89	P = 0.083
		Size	Wλ = 0.24	Wλ = 0.20	Wλ = 0.076
			τ ^2^ = 0.54	τ ^2^ = 0.62	τ ^2^ = 0.86
			P = 0.011*	P = 0.006**	P = 0.001**
		Interaction	**Wλ = 0.32**	Wλ = 0.20	Wλ = 0.38
			τ ^2^ = 0.42	τ ^2^ = 0.14	τ ^2^ = -0.22
			P = 0.045*	P = 0.29	P = 0.85
	Ventral	Ecological factor	**Wλ = 0.34**	Wλ = 0.22	Wλ = 0.16
			τ ^2^ = 0.36	τ ^2^ = 0.071	τ ^2^ = 0.18
			P = 0.050*	P = 0.40	P = 0.29
		Size	Wλ = 0.18	Wλ = 0.17	Wλ = 0.076
			τ ^2^ = 0.67	τ ^2^ = 0.68	τ ^2^ = 0.85
			P = 0.003**	P = 0.0029**	P = 0.001**
		Interaction	**Wλ = 0.29**	Wλ = 0.16	Wλ = 0.15
			τ ^2^ = 0.45	τ ^2^ = 0.20	τ ^2^ = 0.27
			P = 0.043*	P = 0.16	P = 0.27
**Centroid size** (ANOVA)	Dorsal		F = 0.025	F = 0.33	F = 0.73
			R^2^ = 0.0016	R^2^ = 0.039	R^2^ = 0.094
			P = 0.88	P = 0.73	P = 0.50
	Ventral		F = 0.013	F = 0.19	F = 0.33
			R^2^ = 0.0008	R^2^ = 0.023	R^2^ = 0.045
			P = 0.91	P = 0.83	P = 0.73

Level of significance: *<0.05, **<0.01, ***<0.001. Bold indicates relevant results regarding ecological factors. For linear models, we provide the multiple R^2^. For models fitted with penalized likelihood we provide τ ^2^, the multivariate effect size estimated from the permuted data. Wλ:Wilk’s test λ.

When testing the association between ecological factors and skull shape, the estimated pseudo-likelihood of the phylogenetic MANOVA indicated that EB was the best fitted model in all cases (even if the GIC criterion showed small differences): climate and dorsal view (BM: pseudo-likelihood = 562.25, GIC = -1133.31; OU/Lambda: pseudo-likelihood = 562.25, GIC = -1131.31; EB: pseudo-likelihood = 564.35, GIC = -1130.90), climate and ventral view (BM: pseudo-likelihood = 697.52, GIC = -1405.42; OU/Lambda: pseudo-likelihood = 697.52, GIC = -1403.42; EB: pseudo-likelihood = 704.08, GIC = -1404.68), locomotor habitat and dorsal view (BM: pseudo-likelihood = 576.32, GIC = -1140.09; OU/Lambda: pseudo-likelihood = 576.32, GIC = -1138.09; EB: pseudo-likelihood = 578.30, GIC = -1139.40), locomotor habitat and ventral view (BM: pseudo-likelihood = 670.62, GIC = -1359.84; OU/Lambda: pseudo-likelihood = 670.62, GIC = -1357.84; EB: pseudo-likelihood = 676.37, GIC = -1364.19), nychthemeral cycle and dorsal view (BM: pseudo-likelihood = 518.82, GIC = -1054.35; OU: pseudo-likelihood = 518.82, GIC = -1052.35; Lambda: pseudo-likelihood = 519.18, GIC = -1050.22; EB: pseudo-likelihood = 527.03, GIC = -1068.31) and nychthemeral cycle and ventral view (BM: pseudo-likelihood = 618.50, GIC = -1255.56; OU: pseudo-likelihood = 618.50, GIC = -1253.56; Lambda: pseudo-likelihood = 618.51, GIC = -1253.60; EB: pseudo-likelihood = 633.82, GIC = -1272.96).

Climate is the only factor that has a statistically significant influence on shape. In ventral the main effect and the interaction with size are significant, meaning that shape variation related to size cannot be differentiated to shape variation related to climate. In dorsal there is no main effect of climate but the main effect of size and the interaction term are significant meaning that there is a crossover interaction. The effect of size on shape is opposite depending on the climate [48].

**Fig 4:**
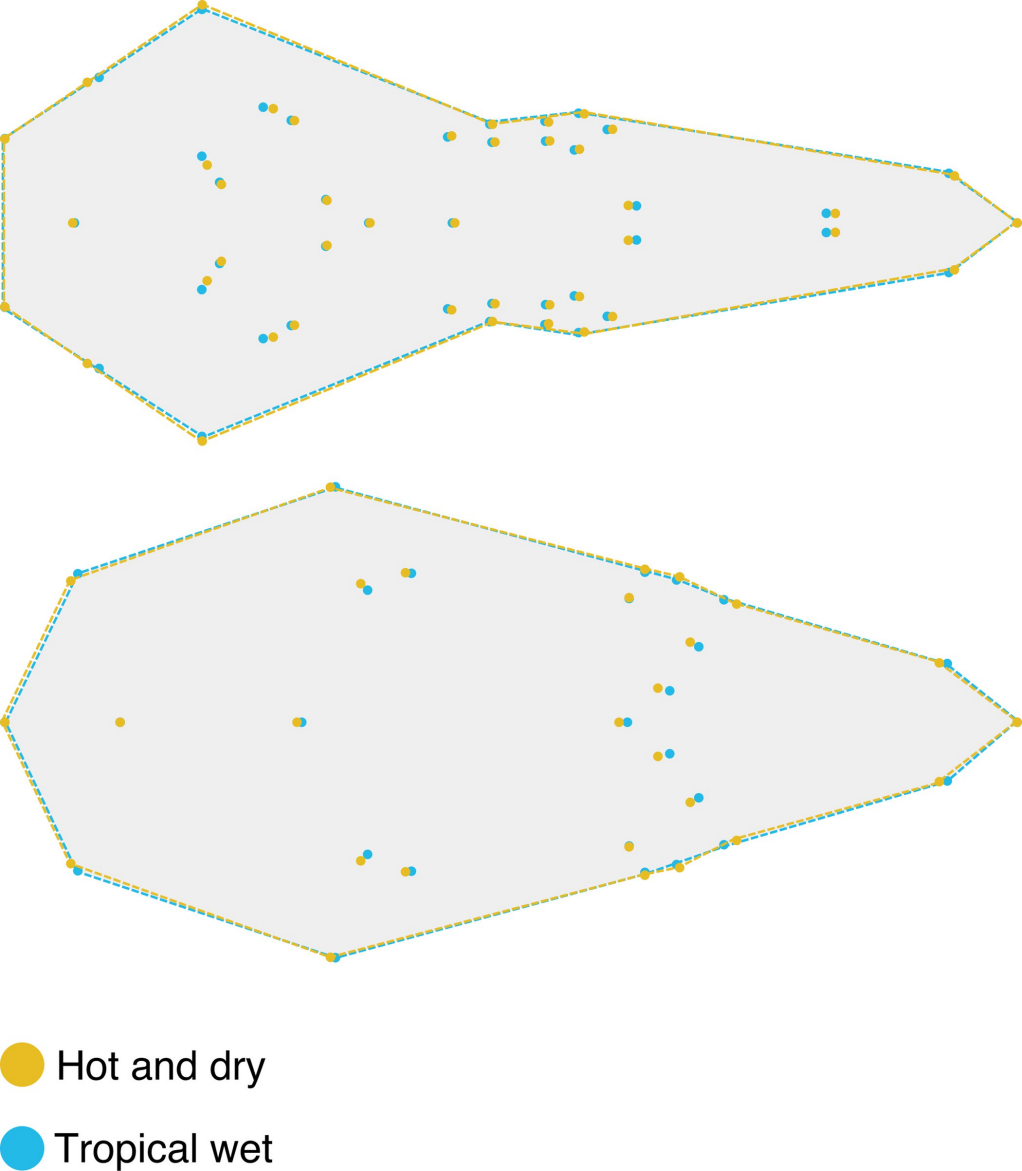
Significant shape changes related to climate. Top: ventral shape; bottom: dorsal shape.

Shape changes related to climate are presented in **Fig 4**. In the ventral cranium species living in “tropical wet” climates have an elongated skull, with a proportionally longer and larger rostra and smaller tympanic bullae compared to species living in “hot and dry” climates. In dorsal cranium species living in “tropical wet” climates have an elongated nasal bone compared to species living in “hot and dry” climates.

## Discussion

### Phylogenetic signal and ecological influence on skull size

No significant phylogenetic signal was detected in skull size (**Table 3**). Size might be conditioned by a factor/factors other than phylogenetic history. However, conversely to what is expected in insular context none of the three tested ecological factors was found to have a significant impact on skull size either (**Table 4**). Hence, either ecological space is not partitioned by size in Nesomyinae, either size may have been driven by other ecological parameters than those tested, as diet. However, diet is highly variable in rodents [43, 49], and more data on the feeding habits of Nesomyinae are needed to test it reliably.

## Phylogenetic signal and ecological influence on skull shape

Skull shape display significant phylogenetic signals (**Table 3**). Skull shape seems to be mainly driven by its evolutionary history. It is confirmed by our PCA results: in dorsal and ventral view the distribution of species in morphological space is congruent with phylogeny (**Figs 2 and 3**). Phylogenetically close species are also morphologically close, such as *Monticolomys koopmani* and *Macrotarsomys bastardi*, or all species belonging to same genus (*Eliurus*, *Brachytarsomys*, *Brachyuromys*, *Nesomys*). In ventral view, *Nesomys rufus* is in the center of the PCA, as it represents the standard ventral shape of a Nesomyinae skull at least based on species present in our dataset. Given that these species are morphologically distinct, their dissimilarities appear rather low compared to their measured molecular distance [8]. The morphological proximity of species could be explained by a partition of resources through different behavior or activity pattern, rather than morphological character displacement. Another hypothesis, not tested in this paper, is that this morphological similarity might be due to convergence, as it has been observed in other endemic mammal lineages such as shrew tenrecs of the genus *Microgale* [50], mouse lemurs of the genus *Microcebus* [51], and long-fingered bats of the genus *Miniopterus* [52].

The evolutionary model that best fits our sample is the early burst model of diversification, and not Brownian motion as it was expected considering the high phylogenetic signal (**Table 3**). Maybe the climate variable provided a stronger explanatory power for skull shape variation, as suggested by Giacomini et al. (2021) [53] who obtained similar results. This mode of evolution usually involves ecological opportunities for concerned species and is typically observed in adaptive radiations [54, 55]. Thus, despite the strong phylogenetic signal displayed by skull shape, it is likely that they are also subjected to adaptation.

Climate was found to significantly influenced shape in ventral cranium and to significantly interact with the influence of size on the shape of dorsal cranium. Two structures are mainly impacted: proportional size of the tympanic bullae and elongation of the rostrum. Species living in arid or semi-desertic environments (“hot and dry”) display wider tympanic bullae than those living in humid environments (“Tropical wet”) (**Fig 4**). A significant influence of climate on this structure is not surprising considering that it is highly sensible to the opening/closing of the environment where the animal lives, as it has been observed in gerbilles and other mammals [56–58]. Associated adaptations could also be related to the margins of the foramen magnum which are neighboring structures of tympanic bullae. Tropical areas are mainly covered with forests while arid areas are more often open environments. Evolving in such different environments requires different mobility abilities. The implication of the foramen magnum in mobility abilities have already been demonstrated in other studies on mammals [59, 60] and is often related to head posture [12, 37, 38]. Variation in the elongation of the rostrum might be related to the differences of available resources in varied environmental conditions. Shape variation associated with the width and length of the rostrum can be related to feeding habits. In rodents, it has been observed that herbivores display longer tooth rows and wider skull and rostrum [61]. However, it is well known that diet is spatially and temporally variable in rodents [49]. As far as we know, most Nesomyines are known to be omnivorous and their diet vary seasonally according to available resources [43]. The influence of climate on shape also strongly interacts with size. Then, climate seems also to affect the allometric pattern of the skull. The differences in allometric patterns between species are, at least in part, explained by differences in the climate of their living environment.

### Temporal perspectives of Nesomyinae skull evolution

We analyzed the extant diversity of Nesomyinae to understand patterns and processes of their diversification since their colonization of Madagascar. However, the current representatives may not depict the maximum diversity of this clade since their arrival, thus biasing our interpretations. Several recent changes must have impacted Nesomyinae shape diversity such as human arrival on the island, natural climatic changes, anthropogenic vicissitudes and the introduction of invasive murids [62–65]. In the Quaternary subfossil record of Madagascar, one notably large-bodied nesomyine is known, *Hypogeomys australis* (Grandidier, 1903), which was notably larger than the only extant member of this genus, *Hypogeomys antimena*, the largest living rodent on the island. In addition, in the subfossil record is the largest known *Nesomys* species, *Nesomys narindaensis* [64], notably bigger than other extant members of this genus, which would include *Nesomys lambertoni* at less than 250 g [43]. Other large-bodied nesomyine species may have gone extinct, but the absence of paleontological data in the Neogene of Madagascar and the scarcity of the Quaternary material hinders any detailed interpretation of past nesomyine diversity and evolution. Among Madagascar endemic mammals, there were giants forms, all extinct in the Late Pleistocene-Holocene, such as *Hypogeomys australis*, of about 2 kg; lemurs, *Archaeoindris* up to 200 kg, and a carnivoran, *Cryptoprocta spelea*, the largest Holocene land predator of Madagascar [63, 66, 67]. Today, the extant *Hypogeomys antimena* only reaches a maximum body mass of slightly greater than 1 kg [43]. The continental African representatives of the Nesomyidae, the closest clades to the Malagasy Nesomyinae [8, 28] all weigh under 100 g, with the exception *Cricetomys* species that reach 2 kg. *Macrotarsomys* and *Monticolomys* are small genera whose molars are morphologically close to fossil genera such as *Protarsomys* and *Notocricetodon*, which date from the lower Miocene of East Africa and may be the ancestors of the Malagasy Nesomyinae [68, 69]. According to molecular clock the crown-group diversification of Nesomyinae occurs around 12.8 Ma and the colonization of Madagascar between that date and 15.6 Ma [28, 70]. *Protarsomys* and *Notocricetodon* (extinct Miocene genera) were small body-size rodents. This may indicate, despite geographical isolation, that at least molar morphology evolved in relative stasis for these genera since the Miocene, or represent an excellent example of convergence on opposite sides of the Mozambique Channel separated by about 20 Ma. The genus *Monticolomys*, which is consistently the sister taxon of *Macrotarsomys* in different molecular phylogenies [8, 28, 71], is morphologically close to *Macrotarsomys* [69]. Such congruence between phylogeny and a low morphological variability interpreted as preservation of an ancestral morphology in some lineages, may be the result of heavy constraints occurring on Nesomyinae skull morphology and no ecological release is readily apparent in this subfamily. Most of all Nesomyinae genera are represented by 1 to 3 species, with the exception of *Eliurus* whose diversity is 13 species [7]. This genus has a relatively homogenous skull shape and includes species ranging from 20 to 100 g. Its success may be related to its specialization towards arboreality during Cenozoic times in Madagascar.

## Conclusion

Nesomyinae skull is a complex structure for which size and shape are not under the same constraints. Skull shape strongly reflects phylogeny, but is also substantially influenced by climate. Skull size revealed to carry a weak phylogenetic signal, as awaited in insular context, but unexpectedly show no adaptive signal regarding ecological factors examined. The large size of Madagascar, its ecological complexity and its particular colonization history of lineages may be associated with unusual types of constraints in island context, preventing the Nesomyinae radiation from displaying strong ecological release.

## Supporting information

S1 Fig Shape PCA individuals. PCA of dorsal (**A**) and ventral (**B**) view symmetric component of all individuals used for analysis.
(PDF)

S2 Fig Allometric slopes of species. Plots of PC1 and PC2 fitted values and the log centroid size in dorsal (**A**) and ventral (**B**) view.
(PDF)

S3 Fig Visualization of the third and fourth axes of the PCA performed on dorsal shape with phylogenetic projection. Colored points represent the morphological average of all individuals of a species. Colors indicate the two principal clades among Nesomyinae. Blue: clade formed by the genus *Brachyuromys*, *Nesomys*, *Macrotarsomys*, *Monticolomys*, and *Hypogeomys*; green: clade formed by the genus *Brachytarsomys*, *Eliurus*, *Gymnuromys*, and *Voalavo*. Warpgrids indicate shape variation along axis with maximum deformation observed at each extremity of the axis.
(PDF)

S4 Fig Visualization of the third and fourth axes of the PCA performed on ventral shape with phylogenetic projection. Colored points represent the morphological average of all individuals of a species. Colors indicate the two principal clades among Nesomyinae. Blue: clade formed by the genus *Brachyuromys*, *Nesomys*, *Macrotarsomys*, *Monticolomys*, and *Hypogeomys*; green: clade formed by the genus *Brachytarsomys*, *Eliurus*, *Gymnuromys*, and *Voalavo*. Warpgrids indicate shape variation along axis with maximum deformation observed at each extremity of the axis.
(PDF)

S1 Table Voucher specimen data. List of used specimens and associated informations. Lines in bold are type specimens (holotypes, syntypes or paratypes).
(DOCX)

S2 Table Dorsal skull landmarks. Descriptions and types of the 27 landmarks used for the dorsal view.
(DOCX)

S3 Table Ventral skull landmarks. Descriptions and types of the 42 landmarks used for the ventral view.
(DOCX)
