# Supplementary figures and images for: Skull morphological evolution in Malagasy endemic Nesomyinae rodents
Source: PLoS One. 2022 Feb 4;17(2):e0263045. doi: 10.1371/journal.pone.0263045 (PMC8815910; doi:10.1371/journal.pone.0263045)

**A**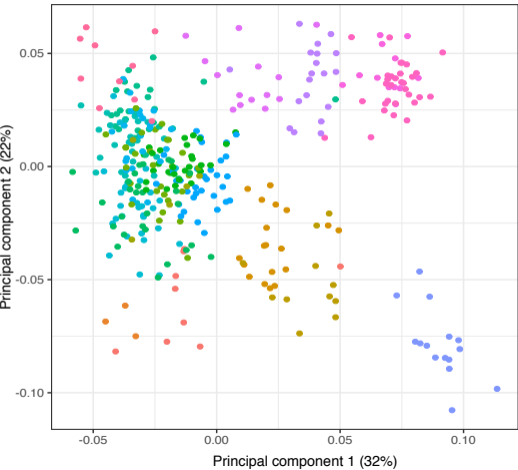**B**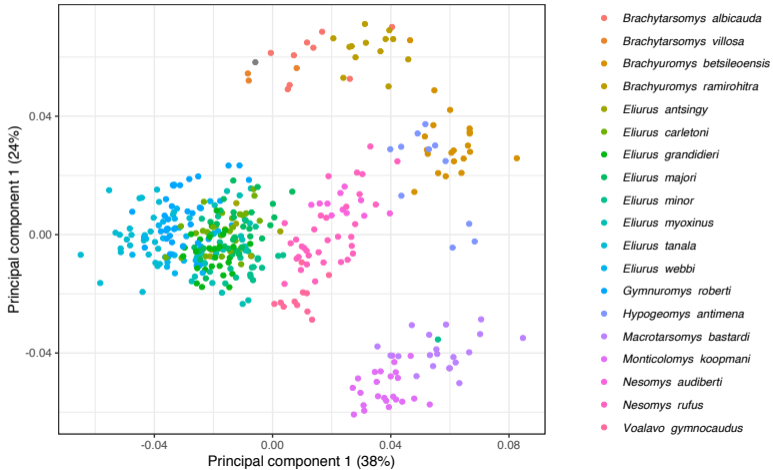

Supplement: S1 Fig — PCA of dorsal (A) and ventral (B) view symmetric component of all individuals used for analysis. (PDF) [file pone.0263045.s001.pdf]

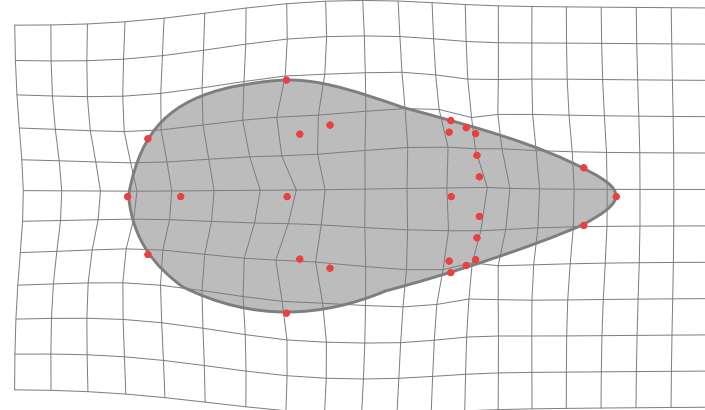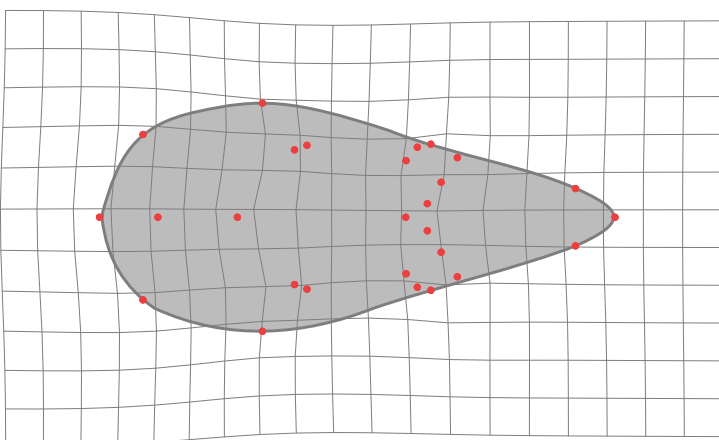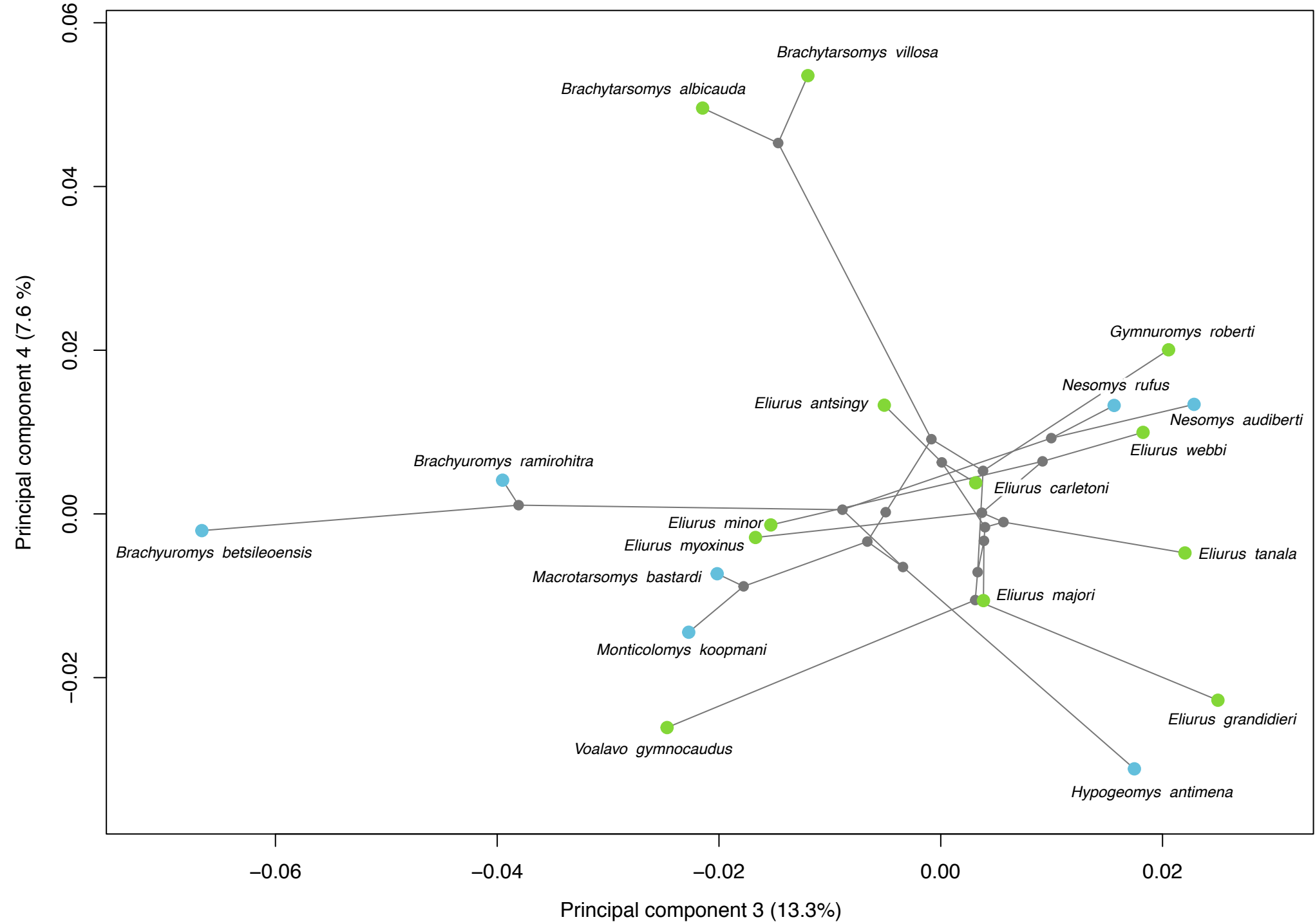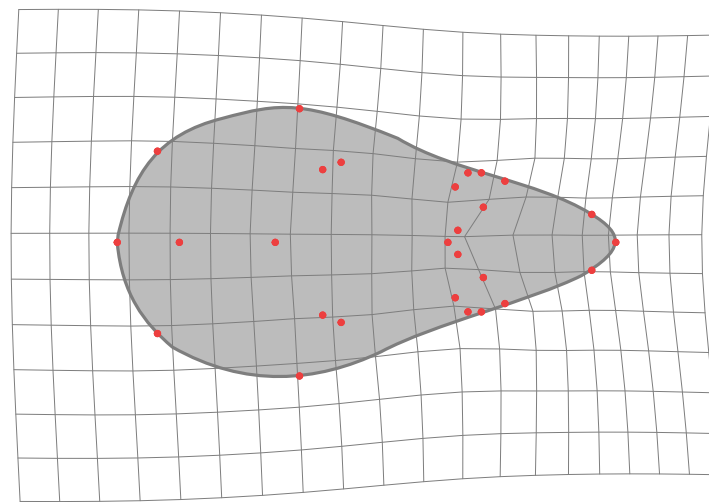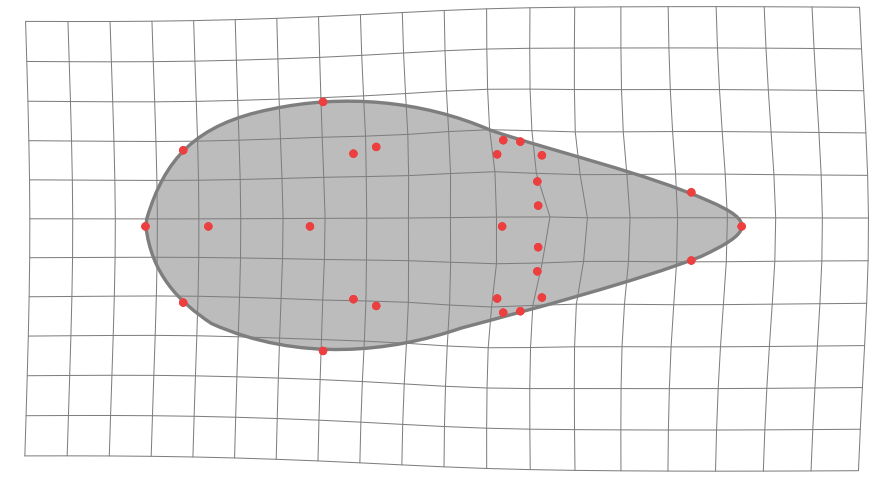

Supplement: S3 Fig — Colored points represent the morphological average of all individuals of a species. Colors indicate the two principal clades among Nesomyinae. Blue: clade formed by the genus Brachyuromys, Nesomys, Macrotarsomys, Monticolomys, and Hypogeomys; green: clade formed by the genus Brachytarsomys, Eliurus, Gymnuromys, and Voalavo. Warpgrids indicate shape variation along axis with maximum deformation observed at each extremity of the axis. (PDF) [file pone.0263045.s003.pdf]

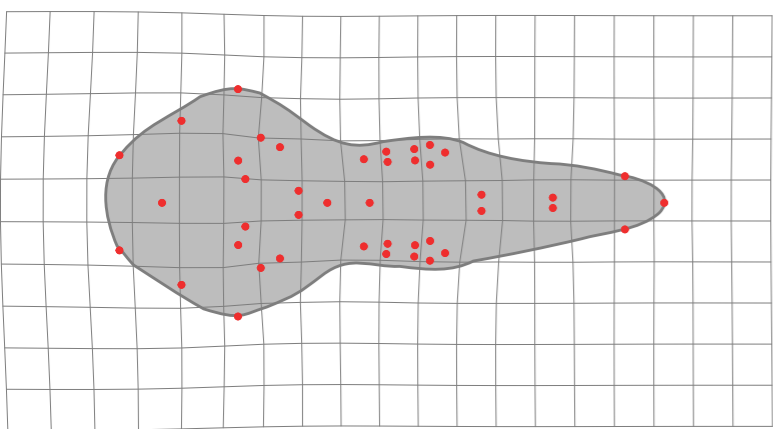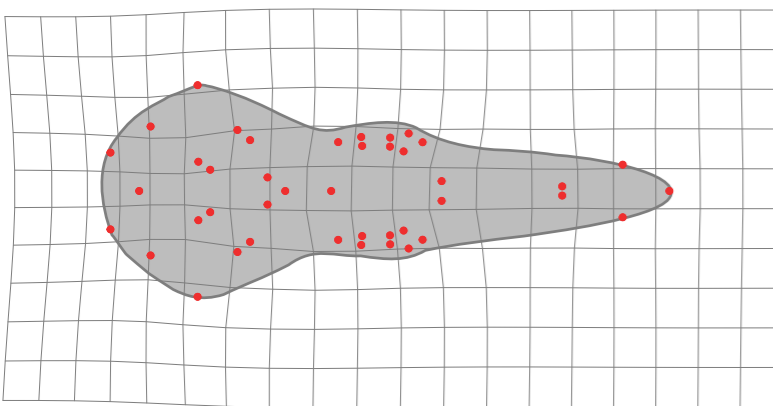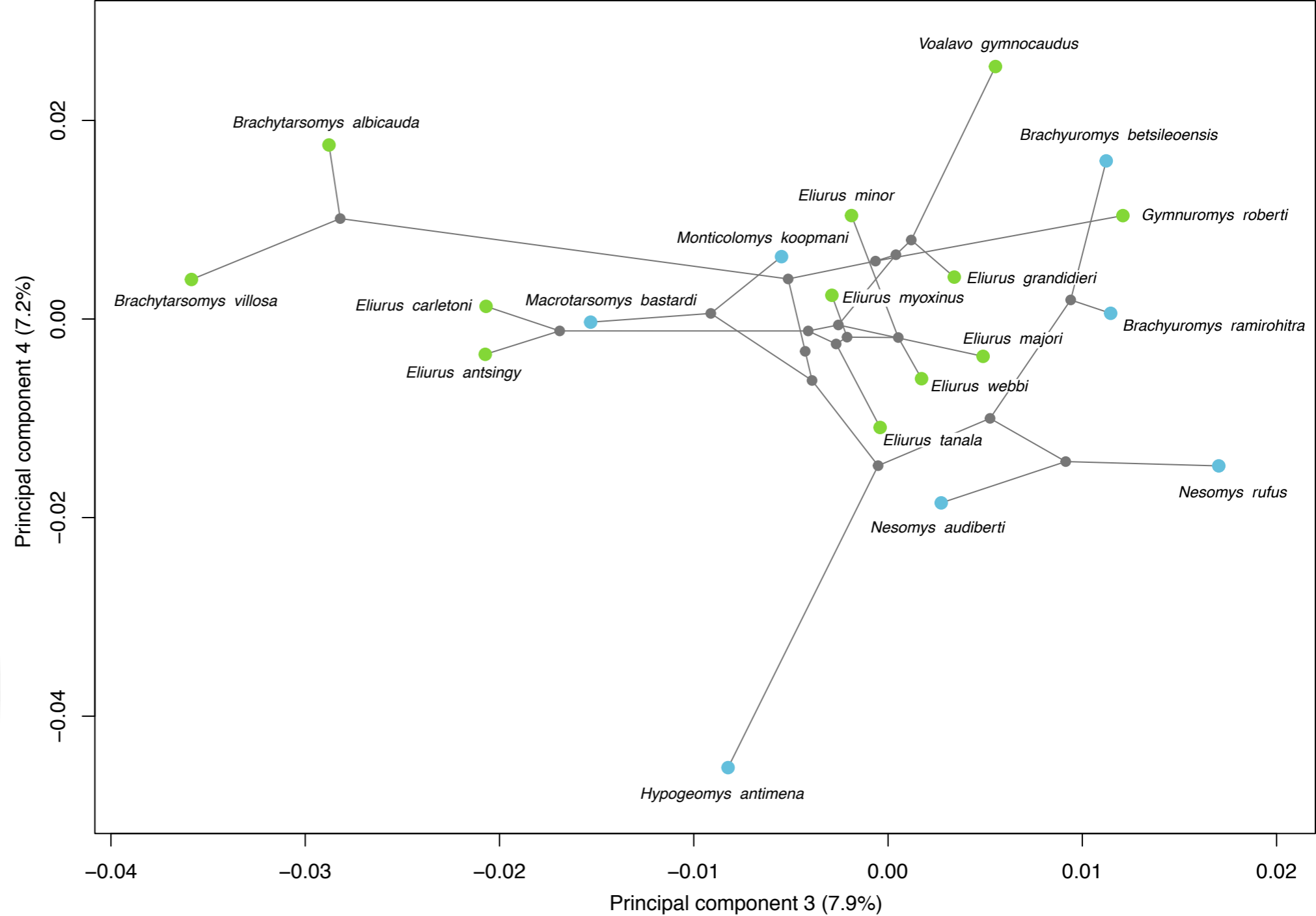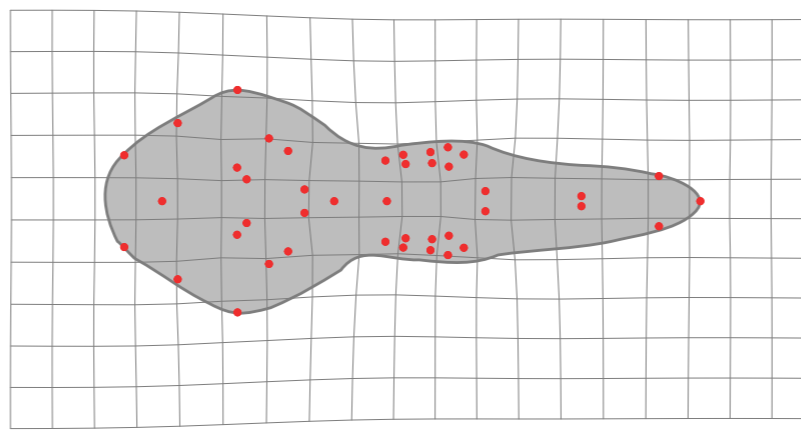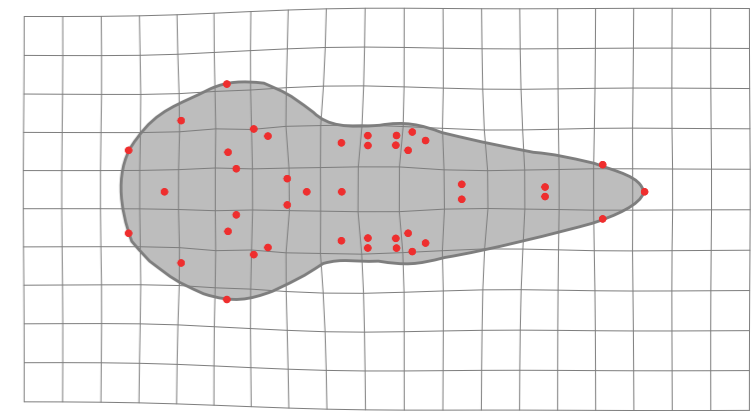

Supplement: S4 Fig — Colored points represent the morphological average of all individuals of a species. Colors indicate the two principal clades among Nesomyinae. Blue: clade formed by the genus Brachyuromys, Nesomys, Macrotarsomys, Monticolomys, and Hypogeomys; green: clade formed by the genus Brachytarsomys, Eliurus, Gymnuromys, and Voalavo. Warpgrids indicate shape variation along axis with maximum deformation observed at each extremity of the axis. (PDF) [file pone.0263045.s004.pdf]
