# Supplementary material for: Skull morphological evolution in Malagasy endemic Nesomyinae rodents
Source: PLoS One. 2022 Feb 4;17(2):e0263045. doi: 10.1371/journal.pone.0263045 (PMC8815910; doi:10.1371/journal.pone.0263045)
Supplement: S2 Fig — Plots of PC1 and PC2 fitted values and the log centroid size in dorsal (A) and ventral (B) view. (PDF) [file pone.0263045.s002.pdf]

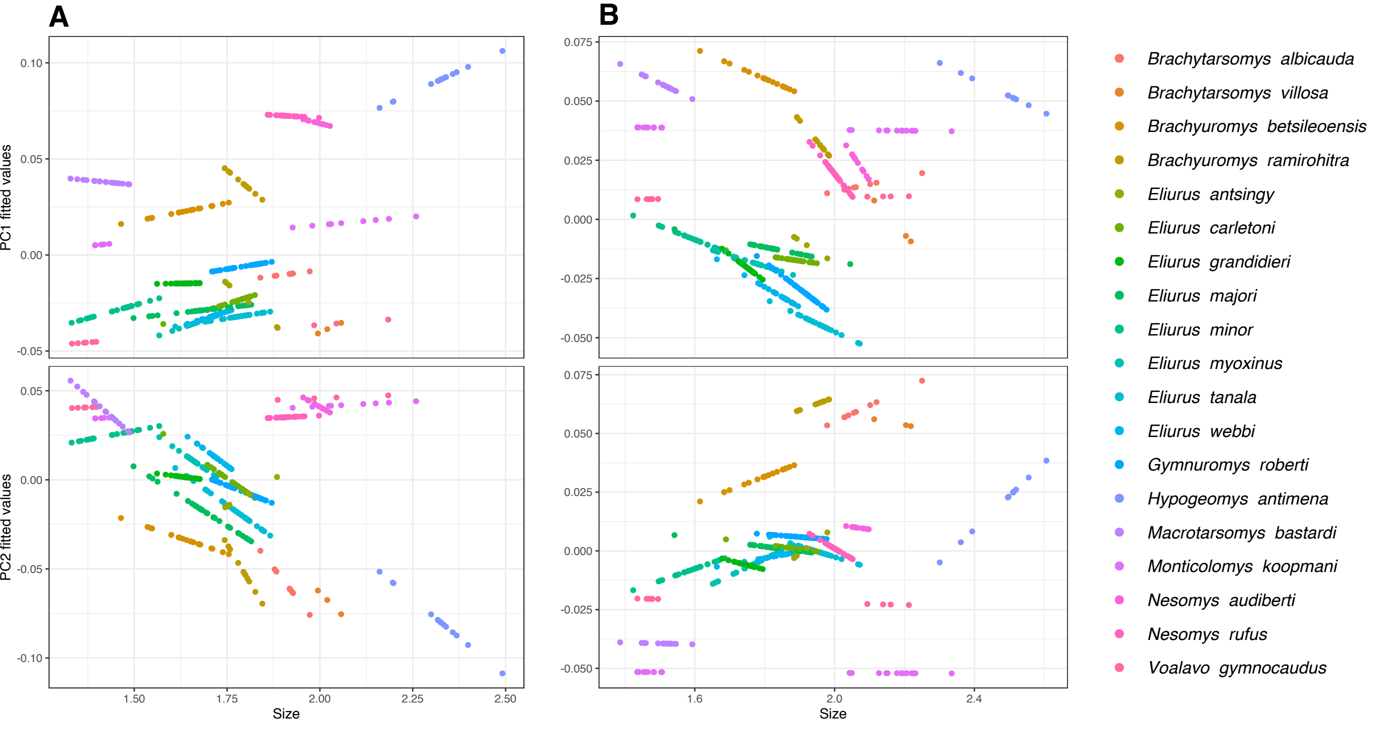

A
B
PC1 fitted values
PC2 fitted values
Size
Size
0.10
0.05
0.00
-0.05
-0.10
0.075
0.050
0.025
0.000
-0.025
-0.050
1.50
1.75
2.00
2.25
2.50
1.6
2.0
2.4
Brachytarsomys albicauda
Brachytarsomys villosa
Brachyuromys betsileoensis
Brachyuromys ramirohitra
Eliurus antsingy
Eliurus carletoni
Eliurus grandidieri
Eliurus majori
Eliurus minor
Eliurus myoxinus
Eliurus tanala
Eliurus webbi
Gymnuromys roberti
Hypogeomys antimena
Macrotarsomys bastardi
Monticolomys koopmani
Nesomys audiberti
Nesomys rufus
Voalavo gymnocaudus
