# Supplementary material for: Skull morphological evolution in Malagasy endemic Nesomyinae rodents
Source: PLoS One. 2022 Feb 4;17(2):e0263045. doi: 10.1371/journal.pone.0263045 (PMC8815910; doi:10.1371/journal.pone.0263045)
Supplement: S1 Table — List of used specimens and associated informations. Lines in bold are type specimens (holotypes, syntypes or paratypes). (DOCX) [file pone.0263045.s005.docx]

**Supplementary Table 1 Nesomyinae rodent geometrics morphometrics**

**Terray et al.**

**Table S1.** List of used specimen and associated informations. Bold = type specimens. X: specimens included either for ventral skull or dorsal or both analyses.

| **Dorsal analysis** | **Ventral analysis** | **Species** | **Specimen number** | **Sex** | **Specimen locality** |
| --- | --- | --- | --- | --- | --- |
| X | X | *Brachytarsomys*  *albicauda* | BMNH 35.1.8.340 | - | Sianaka Forest |
| - | X | *Brachytarsomys albicauda* | ZMB 35353 | - | Toamasina, Sihanaka Forest |
| X | X | *Brachytarsomys albicauda* | ZMB 33375 | - | - |
| **X** | **-** | ***Brachytarsomys albicauda*** | BMNH **75.1.29.13**  **Syntype** | **-** | **Tamantave and Murandava,**  **between** |
| X | - | *Brachytarsomys albicauda* | BMNH 75.1.29.14 | - | Tamantave and Murandava,  between |
| X | X | *Brachytarsomys albicauda* | BMNH 25.12.9.25 | M | Sianaka Forest |
| X | X | *Brachytarsomys albicauda* | BMNH 97.9.1.120 | F | Vinanitelo |
| X | X | *Brachytarsomys albicauda* | BMNH 47.1724 | F | Périnet, near Moramanga |
| X | X | *Brachytarsomys albicauda* | BMNH 47.1726 | F | Imerimandroso, E |
| - | X | *Brachytarsomys albicauda* | UADBA 48029 | - | Antananarivo Province, 8 km SE d'Anjozorobe |
| X | X | *Brachytarsomys albicauda* | FMNH 154055 | F | Mahajanga Province, Anjanaharibe-Sud |
| X | X | *Brachytarsomys albicauda* | MNHN 1897-529 | M | Vinanitelo |
| X | X | *Brachytarsomys villosa* | FMNH 167470 | M | Mahajanga Province, Anjanaharibe-Sud |
| X | X | *Brachytarsomys villosa* | FMNH 167469 | M | Mahajanga Province, Anjanaharibe-Sud |
| **X** | **X** | ***Brachytarsomys villosa*** | **MNHN 1961-221**  **Holotype** | **M** | **Vivarium Tananarive** |
| **X** | **-** | ***Brachyuromys betsileoensis*** | BMNH **80.5.7.2**  **Syntype** | **-** | **Betsileo** |
| X | X | *Brachyuromys betsileoensis* | BMNH 98.3.8.13 | F | Ampitambe |
| X | X | *Brachyuromys betsileoensis* | BMNH 98.3.8.11 | M | Ampitambe |
| X | X | *Brachyuromys betsileoensis* | BMNH 97.9.1.129 | M | Ambohimanana |
| X | X | *Brachyuromys betsileoensis* | FMNH 156228 | F | Fianarantsoa Province, Andringitra |
| X | X | *Brachyuromys betsileoensis* | FMNH 156230 | F | Fianarantsoa Province, Andringitra |
| X | X | *Brachyuromys betsileoensis* | FMNH 156231 | M | Fianarantsoa Province, Andringitra |
| X | X | *Brachyuromys betsileoensis* | FMNH 156232 | F | Fianarantsoa Province, Andringitra |
| X | X | *Brachyuromys betsileoensis* | FMNH 156234 | F | Fianarantsoa Province, Andringitra |
| X | X | *Brachyuromys betsileoensis* | FMNH 156235 | F | Fianarantsoa Province, Andringitra |
| X | X | *Brachyuromys betsileoensis* | FMNH 156237 | F | Fianarantsoa Province, Andringitra |
| X | X | *Brachyuromys betsileoensis* | FMNH 156254 | M | Fianarantsoa Province, Andringitra |
| X | X | *Brachyuromys betsileoensis* | FMNH 156255 | F | Fianarantsoa Province, Andringitra |
| X | X | *Brachyuromys betsileoensis* | FMNH 156256 | F | Fianarantsoa Province, Andringitra |
| X | X | *Brachyuromys betsileoensis* | FMNH 156257 | M | Fianarantsoa Province, Andringitra |
| X | X | *Brachyuromys betsileoensis* | FMNH 156258 | M | Fianarantsoa Province, Andringitra |
| - | X | *Brachyuromys betsileoensis* | FMNH 156259 | M | Fianarantsoa Province, Andringitra |
| X | X | *Brachyuromys betsileoensis* | FMNH 156260 | F | Fianarantsoa Province, Andringitra |
| X | X | *Brachyuromys betsileoensis* | UADBA 48333 | M | Fianarantsoa Province, Andringitra |
| X | X | *Brachyuromys betsileoensis* | UADBA 48316 | M | Fianarantsoa Province, Andringitra |
| - | X | *Brachyuromys betsileoensis* | MNHN 1972-601 | F | Fianarantsoa Province Andringitra |
| X | X | *Brachyuromys betsileoensis* | MNHN 1912-135 | - | Ikongo |
| **X** | **-** | ***Brachyuromys ramirohitra*** | BMNH **97.9.1.133**  **Holotype** | **M** | **Ampitambe** |
| X | X | *Brachyuromys ramirohitra* | BMNH 97.9.1.125 | M | Ampitambe |
| X | X | *Brachyuromys ramirohitra* | BMNH 97.9.1.134 | M | Ampitambe |
| X | X | *Brachyuromys ramirohitra* | BMNH 97.9.1.136 | - | Ampitambe |
| - | X | *Brachyuromys ramirohitra* | BMNH 97.9.1.139 | M | Ampitambe |
| X | X | *Brachyuromys ramirohitra* | BMNH 98.3.8.14 | M | Ampitambe Forest |
| X | X | *Brachyuromys ramirohitra* | BMNH 98.3.8.15 | M | Ampitambe Forest |
| X | X | *Brachyuromys ramirohitra* | BMNH 97.9.1.135 | M | Ampitambe |
| X | X | *Brachyuromys ramirohitra* | BMNH 98.3.8.16 | M | Ampitambe |
| - | X | *Brachyuromys ramirohitra* | FMNH 29469 | F | Fianarantsoa Province, Fandriana |
| X | X | *Brachyuromys ramirohitra* | FMNH 151660 | F | Fianarantsoa Province, Andringitra |
| - | X | *Brachyuromys ramirohitra* | FMNH 167451 | F | Mahajanga Province, Anjanaharibe-Sud |
| X | X | *Brachyuromys ramirohitra* | UADBA 16235 | F | Fandriana-Marolambo |
| X | X | *Brachyuromys ramirohitra* | UADBA 48399 | M | Fianarantsoa Province, Andringitra |
| X | X | *Eliurus antsingy* | FMNH 172721 | M | Toliara Province, Bekopaka |
| X | X | *Eliurus antsingy* | FMNH 175913 | F | Mahajanga Province, Mahabo Forest |
| X | X | *Eliurus antsingy* | FMNH 175909 | F | Mahajanga Province, Ambovonomby Forest |
| X | X | *Eliurus antsingy* | FMNH 175910 | M | Mahajanga Province, Ambovonomby Forest |
| X | X | *Eliurus antsingy* | FMNH 175912 | M | Mahajanga Province, Mahabo Forest |
| X | X | *Eliurus carletoni* | FMNH 178872 | F | Antsiranana Province, Ankavanana Forest |
| - | X | *Eliurus carletoni* | FMNH 178873 | F | Antsiranana Province, Ankavanana Forest |
| X | X | *Eliurus carletoni* | FMNH 178874 | F | Antsiranana Province, Ankavanana Forest |
| X | X | *Eliurus carletoni* | FMNH 178854 | M | Antsiranana Province, Ankavanana Forest |
| X | X | *Eliurus carletoni* | FMNH 178853 | M | Antsiranana Province, Ankavanana Forest |
| X | X | *Eliurus carletoni* | FMNH 169718 | M | Antsiranana Province, Ankarana Forest |
| X | X | *Eliurus carletoni* | FMNH 169719 | F | Antsiranana Province, Ankarana Forest |
| X | X | *Eliurus carletoni* | FMNH 169720 | M | Antsiranana Province, Ankarana Forest |
| X | X | *Eliurus carletoni* | FMNH 173108 | M | Antsiranana Province, Ankarana Forest |
| X | X | *Eliurus carletoni* | FMNH 173109 | F | Antsiranana Province, Ankarana Forest |
| X | X | *Eliurus carletoni* | FMNH 173104 | F | Antsiranana Province, Ankarana Forest |
| X | X | *Eliurus carletoni* | FMNH 173106 | M | Antsiranana Province, Ankarana Forest |
| - | X | *Eliurus carletoni* | FMNH 195973 | F | Antsiranana Province, Ampasibe Maroadabo Forest |
| X | X | *Eliurus carletoni* | FMNH 195975 | M | Antsiranana Province, Ampasibe Maroadabo Forest |
| - | X | *Eliurus carletoni* | FMNH 195976 | F | Antsiranana Province, Ampasibe Maroadabo Forest |
| X | X | *Eliurus carletoni* | FMNH 195831 | F | Antsiranana Province, Ankazomasina Forest |
| X | - | *Eliurus carletoni* | FMNH 195833 | M | Antsiranana Province, Ankazomasina Forest |
| X | X | *Eliurus carletoni* | FMNH 195835 | F | Antsiranana Province, Ankazomasina Forest |
| X | - | *Eliurus carletoni* | FMNH 195839 | M | Antsiranana Province, Ankazomasina Forest |
| X | X | *Eliurus carletoni* | FMNH 195837 | M | Antsiranana Province, Ankazomasina Forest |
| X | X | *Eliurus carletoni* | FMNH 195979 | F | Antsiranana Province, Ampasibe Maroadabo Forest |
| X | - | *Eliurus carletoni* | FMNH 195982 | M | Antsiranana Province, Ambohibory Forest |
| X | X | *Eliurus carletoni* | FMNH 195984 | M | Antsiranana Province, Ambohibory Forest |
| X | X | *Eliurus carletoni* | FMNH 195963 | F | Antsiranana Province, Antsahasolika Forest |
| X | X | *Eliurus carletoni* | FMNH 195965 | F | Antsiranana Province, Antsahasolika Forest |
| X | X | *Eliurus carletoni* | FMNH 195966 | F | Antsiranana Province, Antsahasolika Forest |
| X | X | *Eliurus carletoni* | FMNH 195970 | F | Antsiranana Province, Ambohibe Forest |
| X | X | *Eliurus carletoni* | FMNH 195972 | M | Antsiranana Province, Ambohibe Forest |
| X | X | *Eliurus carletoni* | FMNH 195888 | F | Antsiranana Province, Ambohibory Forest |
| X | X | *Eliurus carletoni* | FMNH 195892 | - | Antsiranana Province, Ambohibory Forest |
| **-** | **X** | ***Eliurus carletoni*** | **FMNH**  **173105**  **Holotype** | **F** | **Antsiranana Province, Ankarana Reserve** |
| - | X | *Eliurus carletoni* | UADBA 46435 | F | Antsiranana Province, Antsahabe Forest |
| X | X | *Eliurus carletoni* | UADBA 46540 | F | Antsiranana Province, Ampondrabe Forest |
| **X** | **X** | ***Eliurus ellermani*** | **BMNH 1947.1623**  **Paratype** | **-** | **Rogez, 13 miles N** |
| X | X | *Eliurus grandidieri* | FMNH 154046 | M | Antsiranana Province, Anjanaharibe-Sud |
| X | X | *Eliurus grandidieri* | FMNH 154047 | M | Antsiranana Province, Anjanaharibe-Sud |
| X | X | *Eliurus grandidieri* | FMNH 154257 | M | Antsiranana Province, Anjanaharibe-Sud |
| X | X | *Eliurus grandidieri* | FMNH 154262 | M | Antsiranana Province, Anjanaharibe-Sud |
| - | X | *Eliurus grandidieri* | FMNH 154265 | F | Antsiranana Province, Anjanaharibe-Sud |
| X | X | *Eliurus grandidieri* | FMNH 154291 | F | Antsiranana Province, Anjanaharibe-Sud |
| X | X | *Eliurus grandidieri* | FMNH 159580 | M | Antsiranana Province, Marojejy |
| X | X | *Eliurus grandidieri* | FMNH 159585 | F | Antsiranana Province, Marojejy |
| X | X | *Eliurus grandidieri* | FMNH 159586 | M | Antsiranana Province, Marojejy |
| X | X | *Eliurus grandidieri* | FMNH 159593 | M | Antsiranana Province, Marojejy |
| - | X | *Eliurus grandidieri* | FMNH 159594 | M | Antsiranana Province, Marojejy |
| X | X | *Eliurus grandidieri* | FMNH 159604 | M | Antsiranana Province, Marojejy |
| X | X | *Eliurus grandidieri* | FMNH 159608 | M | Antsiranana Province, Marojejy |
| X | X | *Eliurus grandidieri* | FMNH 159609 | M | Antsiranana Province, Marojejy |
| X | X | *Eliurus grandidieri* | FMNH 159610 | F | Antsiranana Province, Marojejy |
| X | X | *Eliurus grandidieri* | FMNH 173269 | M | Antsiranana Province, Marojejy |
| - | X | *Eliurus grandidieri* | FMNH 167459 | F | Mahajanga Province, Anjanaharibe-Sud |
| X | X | *Eliurus grandidieri* | FMNH 167461 | M | Mahajanga Province, Anjanaharibe-Sud |
| X | X | *Eliurus grandidieri* | FMNH 167460 | M | Mahajanga Province, Anjanaharibe-Sud |
| X | X | *Eliurus grandidieri* | FMNH 167462 | F | Mahajanga Province, Anjanaharibe-Sud |
| X | X | *Eliurus grandidieri* | FMNH 167464 | M | Mahajanga Province, Anjanaharibe-Sud |
| X | X | *Eliurus grandidieri* | FMNH 167463 | F | Mahajanga Province, Anjanaharibe-Sud |
| X | X | *Eliurus grandidieri* | FMNH 173270 | F | Antsiranana Province, Marojejy |
| X | X | *Eliurus grandidieri* | FMNH 167466 | M | Mahajanga Province, Anjanaharibe-Sud |
| X | X | *Eliurus grandidieri* | FMNH 167465 | M | Mahajanga Province, Anjanaharibe-Sud |
| X | X | *Eliurus grandidieri* | FMNH 166161 | F | Antananarivo Province, Antsahabe Forest |
| X | X | *Eliurus grandidieri* | FMNH 166209 | M | Antsiranana Province, Antanambao |
| X | X | *Eliurus grandidieri* | FMNH 166210 | F | Antsiranana Province, Antanambao |
| X | X | *Eliurus grandidieri* | FMNH 159467 | F | Antananarivo Province, Anjozorobe |
| X | X | *Eliurus grandidieri* | FMNH 159468 | F | Antananarivo Province, Anjozorobe |
| **X** | **X** | ***Eliurus grandidieri*** | **FMNH 154048**  **Holotype** | **F** | **Antsiranana, Anjanaharibe-Sud** |
| X | X | *Eliurus grandidieri* | FMNH 159579 | M | Antsiranana Province, Marojejy |
| X | X | *Eliurus grandidieri* | UADBA 32477 | - | Mahajanga Province, Anjanaharibe-Sud |
| **X** | **X** | ***Eliurus majori*** | **BMNH 97.9.1.147**  **Holotype** | **M** | **Ambohinitromito** |
| X | X | *Eliurus majori* | FMNH 154535 | F | Antsiranana Province, Montagne d’Ambre |
| - | X | *Eliurus majori* | FMNH 154536 | M | Antsiranana Province, Montagne d’Ambre |
| X | X | *Eliurus majori* | FMNH 154538 | F | Antsiranana Province, Montagne d’Ambre |
| - | X | *Eliurus majori* | FMNH 154537 | M | Antsiranana Province, Montagne d’Ambre |
| X | X | *Eliurus majori* | FMNH 154539 | F | Antsiranana Province, Montagne d’Ambre |
| - | X | *Eliurus majori* | FMNH 154603 | F | Antsiranana Province, Montagne d’Ambre |
| X | X | *Eliurus majori* | FMNH 154604 | F | Antsiranana Province, Montagne d’Ambre |
| X | X | *Eliurus majori* | FMNH 154605 | F | Antsiranana Province, Montagne d’Ambre |
| X | X | *Eliurus majori* | FMNH 154606 | M | Antsiranana Province, Montagne d’Ambre |
| X | X | *Eliurus majori* | FMNH 154607 | M | Antsiranana Province, Montagne d’Ambre |
| X | X | *Eliurus majori* | FMNH 154608 | F | Antsiranana Province, Montagne d’Ambre |
| X | X | *Eliurus majori* | FMNH 154609 | F | Antsiranana Province, Montagne d’Ambre |
| X | X | *Eliurus majori* | FMNH 154610 | M | Antsiranana Province, Montagne d’Ambre |
| X | X | *Eliurus majori* | FMNH 154611 | F | Antsiranana Province, Montagne d’Ambre |
| X | X | *Eliurus majori* | FMNH 154612 | F | Antsiranana Province, Montagne d’Ambre |
| X | X | *Eliurus majori* | FMNH 154613 | F | Antsiranana Province, Montagne d’Ambre |
| X | X | *Eliurus majori* | FMNH 154614 | - | Antsiranana Province, Montagne d’Ambre |
| X | X | *Eliurus majori* | FMNH 154615 | M | Antsiranana Province, Montagne d’Ambre |
| X | X | *Eliurus majori* | FMNH 159624 | M | Antsiranana Province, Marojejy |
| X | X | *Eliurus majori* | FMNH 159625 | M | Antsiranana Province, Marojejy |
| X | X | *Eliurus majori* | FMNH 159626 | F | Antsiranana Province, Marojejy |
| X | X | *Eliurus majori* | FMNH 159627 | M | Antsiranana Province, Marojejy |
| X | X | *Eliurus majori* | FMNH 159628 | F | Antsiranana Province, Marojejy |
| X | X | *Eliurus majori* | FMNH 159629 | M | Antsiranana Province, Marojejy |
| X | X | *Eliurus majori* | FMNH 159630 | M | Antsiranana Province, Marojejy |
| X | X | *Eliurus majori* | FMNH 159637 | M | Antsiranana Province, Marojejy |
| X | X | *Eliurus majori* | FMNH 159709 | M | Antsiranana Province, Marojejy |
| X | X | *Eliurus majori* | FMNH 159711 | M | Antsiranana Province, Marojejy |
| X | X | *Eliurus majori* | FMNH 159712 | F | Antsiranana Province, Marojejy |
| X | - | *Eliurus majori* | FMNH 159713 | F | Antsiranana Province, Marojejy |
| - | X | *Eliurus majori* | UADBA 30059 | - | Antananarivo Province, Anjozorobe |
| X | X | *Eliurus majori* | UADBA 30194 | - | Antananarivo Province, Ambohitantely |
| **X** | **X** | ***Eliurus minor*** | **BMNH 97.9.1.153**  **Holotype** | **M** | **Ampitambe** |
| X | X | *Eliurus minor* | BMNH 47.1619 | M | Imerimandroso, Lac Alaotra |
| - | X | *Eliurus minor* | BMNH 35.1.8.346 | F | Vondrozo |
| X | X | *Eliurus minor* | BMNH 47.1617 | F | Périnet, near Moramanga |
| X | X | *Eliurus minor* | MNHN 1987.107 | F | Toamasina Province, Anandrivola Forest |
| X | X | *Eliurus minor* | BMNH 47.1615 | M | Périnet, near Moramanga |
| X | X | *Eliurus minor* | FMNH 151676 | F | Fianarantsoa Province, Andringitra |
| X | X | *Eliurus minor* | FMNH 151678 | M | Fianarantsoa Province, Andringitra |
| X | X | *Eliurus minor* | FMNH 151679 | F | Fianarantsoa Province, Andringitra |
| X | X | *Eliurus minor* | FMNH 151734 | F | Fianarantsoa Province, Andringitra |
| X | X | *Eliurus minor* | FMNH 151735 | M | Fianarantsoa Province, Andringitra |
| X | X | *Eliurus minor* | FMNH 151736 | F | Fianarantsoa Province, Andringitra |
| X | X | *Eliurus minor* | FMNH 151737 | M | Fianarantsoa Province, Andringitra |
| X | X | *Eliurus minor* | FMNH 151668 | M | Fianarantsoa Province, Andringitra |
| - | X | *Eliurus minor* | FMNH 151669 | M | Fianarantsoa Province, Andringitra |
| X | X | *Eliurus minor* | FMNH 151670 | M | Fianarantsoa Province, Andringitra |
| X | X | *Eliurus minor* | FMNH 151671 | F | Fianarantsoa Province, Andringitra |
| X | X | *Eliurus minor* | FMNH 151672 | F | Fianarantsoa Province, Andringitra |
| X | X | *Eliurus minor* | FMNH 151674 | M | Fianarantsoa Province, Andringitra |
| X | X | *Eliurus minor* | FMNH 156618 | F | Toliara Province, Andohahela |
| X | X | *Eliurus minor* | FMNH 156619 | F | Toliara Province, Andohahela |
| X | X | *Eliurus minor* | FMNH 156621 | M | Toliara Province, Andohahela |
| X | X | *Eliurus minor* | FMNH 156622 | F | Toliara Province, Andohahela |
| X | X | *Eliurus minor* | FMNH 156623 | M | Toliara Province, Andohahela |
| X | X | *Eliurus minor* | FMNH 156624 | M | Toliara Province, Andohahela |
| X | X | *Eliurus minor* | UADBA 48089 | - | Antananarivo Province, Anjozorobe |
| X | X | *Eliurus minor* | UADBA 48088 | - | Antananarivo Province, Anjozorobe |
| X | X | *Eliurus myoxinus* | BMNH 49.1606 | F | Bevilany, 5 miles E of, Ambovombe-Fort Dauphin Rd |
| X | X | *Eliurus myoxinus* | BMNH 47.1601 | F | Bevilany, 5 miles E of, Ambovombe-Fort Dauphin Rd |
| X | X | *Eliurus myoxinus* | BMNH 47.1607 | F | Bevilany, 5 miles E of, Ambovombe-Fort Dauphin Rd |
| X | X | *Eliurus myoxinus* | BMNH 47.1603 | F | Bevilany, 5 miles E of, Ambovombe-Fort Dauphin Rd |
| X | - | *Eliurus myoxinus* | BMNH 47.1610 | M | Tulear, forest 35 miles E of |
| X | X | *Eliurus myoxinus* | BMNH 47.1609 | F | Beroboka, 40 miles N of Morondava |
| X | X | *Eliurus myoxinus* | FMNH 172606 | F | Antsiranana Province, Marojejy |
| X | X | *Eliurus myoxinus* | FMNH 172607 | F | Antsiranana Province, Marojejy |
| X | X | *Eliurus myoxinus* | FMNH 172608 | M | Antsiranana Province, Marojejy |
| - | X | *Eliurus myoxinus* | FMNH 172609 | F | Antsiranana Province, Marojejy |
| X | X | *Eliurus myoxinus* | FMNH 172610 | M | Antsiranana Province, Marojejy |
| X | X | *Eliurus myoxinus* | FMNH 172611 | F | Antsiranana Province, Marojejy |
| X | X | *Eliurus myoxinus* | FMNH 172612 | F | Antsiranana Province, Marojejy |
| X | X | *Eliurus myoxinus* | FMNH 172613 | M | Antsiranana Province, Marojejy |
| X | X | *Eliurus myoxinus* | FMNH 172664 | M | Antsiranana Province, Daraina |
| - | X | *Eliurus myoxinus* | FMNH 173207 | M | Antsiranana Province, Marojejy |
| X | X | *Eliurus myoxinus* | FMNH 173209 | F | Antsiranana Province, Marojejy |
| X | X | *Eliurus myoxinus* | FMNH 173211 | M | Antsiranana Province, Marojejy |
| X | X | *Eliurus myoxinus* | FMNH 173212 | M | Antsiranana Province, Marojejy |
| X | - | *Eliurus myoxinus* | FMNH 173213 | M | Antsiranana Province, Marojejy |
| X | X | *Eliurus myoxinus* | FMNH 173214 | M | Antsiranana Province, Marojejy |
| X | X | *Eliurus myoxinus* | FMNH 161579 | M | Toliara Province, Analavelona Forest |
| X | X | *Eliurus myoxinus* | FMNH 161580 | F | Toliara Province, Analavelona Forest |
| X | X | *Eliurus myoxinus* | FMNH 161581 | M | Toliara Province, Analavelona Forest |
| - | X | *Eliurus myoxinus* | FMNH 161582 | M | Toliara Province, Analavelona Forest |
| X | X | *Eliurus myoxinus* | FMNH 161583 | F | Toliara Province, Analavelona Forest |
| X | X | *Eliurus myoxinus* | UADBA 30172 | - | Toliara Province, Kirindy Mité |
| X | X | *Eliurus myoxinus* | UADBA 46998 | - | Mahajanga Province, Marotandrano |
| X | X | *Eliurus tanala* | BMNH 47.15.67 | F | Périnet, near Moramanga |
| X | X | *Eliurus tanala* | BMNH 47.15.60 | M | Périnet, near Moramanga |
| X | X | *Eliurus tanala* | BMNH 47.15.66 | F | Périnet, near Moramanga |
| X | X | *Eliurus tanala* | BMNH 47.15.68 | F | Périnet, near Moramanga |
| **X** | **X** | ***Eliurus tanala*** | **BMNH 97.9.1.154**  **Holotype** | **M** | **Vinanitelo** |
| X | X | *Eliurus tanala* | BMNH 36.11.2.1 | F | Sianaka Forest |
| X | X | *Eliurus ellermani* | FMNH 167515 | M | Antsiranana Province, Betaolana Forest |
| X | X | *Eliurus ellermani* | FMNH 167516 | F | Antsiranana Province, Betaolana Forest |
| - | X | *Eliurus ellermani* | FMNH 167517 | M | Antsiranana Province, Betaolana Forest |
| X | X | *Eliurus ellermani* | FMNH 167518 | F | Antsiranana Province, Betaolana Forest |
| X | X | *Eliurus ellermani* | FMNH 167519 | F | Antsiranana Province, Betaolana Forest |
| X | X | *Eliurus tanala* | FMNH 170833 | M | Fianarantsoa Province, Andrambovato |
| X | X | *Eliurus tanala* | FMNH 170834 | F | Fianarantsoa Province, Andrambovato |
| X | X | *Eliurus tanala* | FMNH 170835 | M | Fianarantsoa Province, Andrambovato |
| - | X | *Eliurus tanala* | FMNH 170836 | M | Fianarantsoa Province, Vinantelo Forest |
| X | X | *Eliurus tanala* | FMNH 170837 | F | Fianarantsoa Province, Vinantelo Forest |
| X | X | *Eliurus tanala* | FMNH 170838 | F | Fianarantsoa Province, Vinantelo Forest |
| X | X | *Eliurus tanala* | FMNH 170839 | M | Fianarantsoa Province, Vinantelo Forest |
| X | X | *Eliurus tanala* | FMNH 170840 | M | Fianarantsoa Province, Vinantelo Forest |
| X | X | *Eliurus tanala* | FMNH 170841 | F | Fianarantsoa Province, Vinantelo Forest |
| X | X | *Eliurus tanala* | FMNH 170824 | M | Fianarantsoa Province, Ranomafana |
| X | - | *Eliurus tanala* | FMNH 170825 | F | Fianarantsoa Province, Ranomafana |
| X | X | *Eliurus tanala* | FMNH 170826 | F | Fianarantsoa Province, Ranomafana |
| X | X | *Eliurus tanala* | FMNH 170827 | F | Fianarantsoa Province, Ranomafana |
| X | X | *Eliurus tanala* | FMNH 170829 | F | Fianarantsoa Province, Ranomafana |
| X | X | *Eliurus tanala* | FMNH 170831 | - | Fianarantsoa Province, Ranomafana |
| X | X | *Eliurus tanala* | FMNH 170832 | M | Fianarantsoa Province, Ranomafana |
| X | - | *Eliurus tanala* | FMNH 156515 | F | Toliara Province, Andohahela |
| - | - | *Eliurus tanala* | FMNH 156518 | M | Toliara Province, Andohahela |
| X | X | *Eliurus tanala* | FMNH 156519 | F | Toliara Province, Andohahela |
| X | X | *Eliurus tanala* | FMNH 156520 | F | Toliara Province, Andohahela |
| - | X | *Eliurus tanala* | FMNH 156521 | F | Toliara Province, Andohahela |
| X | X | *Eliurus tanala* | FMNH 156528 | M | Toliara Province, Andohahela |
| X | X | *Eliurus tanala* | FMNH 156531 | M | Toliara Province, Andohahela |
| X | X | *Eliurus tanala* | FMNH 156631 | M | Toliara Province, Andohahela |
| X | X | *Eliurus tanala* | FMNH 209157 | F | Toamasina Province, Maromizaha Forest |
| X | X | *Eliurus tanala* | UADBA 32565 | M | Toamasina Province, Torotorofotsy |
| X | X | *Eliurus tanala* | UADBA 33254 | - | Toamasina Province, Ankerana |
| X | X | *Eliurus webbi* | FMNH 159721 | F | Antsiranana Province, Marojejy |
| X | X | *Eliurus webbi* | FMNH 159720 | F | Antsiranana Province, Marojejy |
| X | X | *Eliurus webbi* | FMNH 159718 | F | Antsiranana Province, Marojejy |
| X | X | *Eliurus webbi* | FMNH 159722 | M | Antsiranana Province, Marojejy |
| X | X | *Eliurus webbi* | FMNH 172617 | F | Antsiranana Province, Marojejy, Marojejy |
| X | X | *Eliurus webbi* | FMNH 173215 | M | Antsiranana Province, Marojejy, Marojejy |
| X | X | *Eliurus webbi* | FMNH 173224 | M | Antsiranana Province, Marojejy, Marojejy |
| X | X | *Eliurus webbi* | FMNH 151742 | F | Fianarantsoa Province, Andringitra |
| X | X | *Eliurus webbi* | FMNH 151884 | M | Fianarantsoa Province, Andringitra |
| X | X | *Eliurus webbi* | FMNH 151888 | M | Fianarantsoa Province, Andringitra |
| X | X | *Eliurus webbi* | FMNH 151892 | M | Fianarantsoa Province, Andringitra |
| X | X | *Eliurus webbi* | FMNH 151893 | F | Fianarantsoa Province, Andringitra |
| X | X | *Eliurus webbi* | FMNH 151894 | M | Fianarantsoa Province, Andringitra |
| X | X | *Eliurus webbi* | FMNH 151895 | M | Fianarantsoa Province, Andringitra |
| X | X | *Eliurus webbi* | FMNH 151680 | X | Fianarantsoa Province, Andringitra |
| X | - | *Eliurus webbi* | FMNH 151681 | M | Fianarantsoa Province, Andringitra |
| X | X | *Eliurus webbi* | FMNH 151682 | F | Fianarantsoa Province, Andringitra |
| X | X | *Eliurus webbi* | FMNH 151683 | M | Fianarantsoa Province, Andringitra |
| - | X | *Eliurus webbi* | FMNH 151684 | M | Fianarantsoa Province, Andringitra |
| X | X | *Eliurus webbi* | FMNH 151686 | M | Fianarantsoa Province, Andringitra |
| X | X | *Eliurus webbi* | UADBA 10201 | M | Toliara Province, Analalava |
| X | X | *Eliurus webbi* | UADBA 10204 | M | Toliara Province, Nahampoana |
| X | X | *Eliurus webbi* | FMNH 159719 | M | Antsiranana Province, Marojejy |
| - | X | *Eliurus webbi* | FMNH 154036 - | F | Antsiranana Province, Anjanaharibe-Sud |
| **X** | **X** | ***Eliurus webbi*** | **BMNH 47.1576**  **Holotype** | **M** | **Farafangana, 20 miles S of** |
| X | X | *Eliurus webbi* | BMNH 47.15.93 | F | 8 km from Rantabé, east of Antongil Bay |
| X | X | *Eliurus webbi* | BMNH 47.15.96 | F | Fianarantsoa Province, SE of Ivohibé |
| X | X | *Eliurus webbi* | BMNH 47.15.98 | F | Fianarantsoa Province, SE of Ivohibé |
| X | X | *Eliurus webbi* | BMNH 47.15.79 | F | Fianarantsoa Province, SE of Ivohibé |
| X | X | *Eliurus webbi* | **BMNH 47.15.82 Paratype** | **F** | **SE of Farafangana** |
| X | X | *Eliurus webbi* | **BMNH 47.15.75 Paratype** | **M** | **SE of Farafangana** |
| X | X | *Gymnuromys roberti* | BMNH 97.9.1.142 | F | Ampitambe |
| X | X | *Gymnuromys roberti* | BMNH 97.9.1.143 | M | Ampitambe |
| X | X | *Gymnuromys roberti* | BMNH 97.9.1.141 | F | Ampitambe |
| X | X | *Gymnuromys roberti* | BMNH 98.3.8.10 | F | Ampitambe |
| X | X | *Gymnuromys roberti* | BMNH 74.767 | M | Ampitambe |
| X | X | *Gymnuromys roberti* | BMNH 98.3.8.8. | F | Ampitambe |
| X | X | *Gymnuromys roberti* | BMNH 98.3.8.9 | F | Ampitambe |
| X | X | *Gymnuromys roberti* | MNHN 1992-215 | - | Ampitambe |
| X | X | *Gymnuromys roberti* | BMNH 97.9.1.144 | M | Ampitambe |
| X | X | *Gymnuromys roberti* | FMNH 226061 | F | Toamasina Province, Ambalafary Forest |
| X | X | *Gymnuromys roberti* | FMNH 194700 | F | Fianarantsoa Province, Andohabatotany Forest |
| X | X | *Gymnuromys roberti* | FMNH 194699 | F | Fianarantsoa Province, Andohabatotany Forest |
| X | X | *Gymnuromys roberti* | FMNH 194716 | M | Fianarantsoa Province, Vohipia Forest |
| X | X | *Gymnuromys roberti* | FMNH 156614 | F | Toliara Province, Andohahela |
| X | X | *Gymnuromys roberti* | FMNH 167471 | M | Mahajanga Province, Anjanaharibe-Sud |
| X | X | *Gymnuromys roberti* | FMNH 178780 | M | Fianarantsoa Province, Andranomena |
| X | X | *Gymnuromys roberti* | FMNH 170844 | F | Fianarantsoa Province, Vinantelo Forest |
| - | X | *Gymnuromys roberti* | FMNH 178717 | M | Fianarantsoa Province, Befotaka |
| X | X | *Gymnuromys roberti* | FMNH 170843 | F | Fianarantsoa Province, Andrambovato |
| X | X | *Gymnuromys roberti* | FMNH 151694 | M | Fianarantsoa Province, Andringitra |
| - | X | *Gymnuromys roberti* | FMNH 5632 | F | - |
| X | X | *Gymnuromys roberti* | FMNH 151695 | F | Fianarantsoa Province, Andringitra |
| X | X | *Gymnuromys roberti* | FMNH 161909 | M | Fianarantsoa Province, Angodongodona |
| X | X | *Gymnuromys roberti* | FMNH 162092 | F | Fianarantsoa Province, Ivohibe |
| X | X | *Gymnuromys roberti* | FMNH 162093 | F | Fianarantsoa Province, Ivohibe |
| X | X | *Gymnuromys roberti* | FMNH 162094 | F | Fianarantsoa Province, Angodongodona |
| X | X | *Gymnuromys roberti* | FMNH 162095 | M | Fianarantsoa Province, Angodongodona |
| X | X | *Gymnuromys roberti* | FMNH 159724 | F | Antsiranana Province, Marojejy |
| X | X | *Gymnuromys roberti* | FMNH 188755 | F | Antananarivo Province, Anjozorobe |
| X | X | *Gymnuromys roberti* | FMNH 166165 | F | Antananarivo Province, Tsinjoarivo |
| X | X | *Gymnuromys roberti* | FMNH 188675 | F | Antananarivo Province, Anjozorobe |
| X | X | *Gymnuromys roberti* | FMNH 172618 | F | Antsiranana Province, Marojejy |
| X | X | *Gymnuromys roberti* | FMNH 154056 | M | Antsiranana Province, Anjanaharibe-Sud |
| X | X | *Gymnuromys roberti* | UADBA 48050 | - | Antananarivo Province, Anjozorobe |
| - | X | *Gymnuromys roberti* | UADBA 30173 | - | - |
| **X** | **X** | ***Gymnuronys roberti*** | **BMNH 97.9.1.140**  **Holotype** | **F** | **Ampitambe** |
| **X** | **X** | ***Hypogeomys antimena*** | **ZMB 5405**  **Syntype** | **-** | **West coast of Madagascar** |
| X | X | *Hypogeomys antimena* | FMNH 161570 | M | Toliara Province, Kirindy Forest |
| X | X | *Hypogeomys antimena* | FMNH 161571 | F | Toliara Province, Kirindy Forest |
| X | X | *Hypogeomys antimena* | FMNH 151994 | M | Toliara Province, Kirindy Forest |
| X | X | *Hypogeomys antimena* | FMNH 154635 | F | Toliara Province, Kirindy Forest |
| X | X | *Hypogeomys antimena* | FMNH 161569 | M | Toliara Province, Kirindy Forest |
| X | - | *Hypogeomys antimena* | FMNH 154636 | M | Toliara Province, Kirindy Forest |
| X | - | *Hypogeomys antimena* | FMNH 151995 | - | Toliara Province, Kirindy Forest |
| X | - | *Hypogeomys antimena* | MNHN 1962-2023 | - | Fianarantsoa Province, Midongy-Sud |
| X | X | *Hypogeomys antimena* | MNHN 1961-222 | F | Beroboka |
| **X** | **X** | ***Hypogeomys antimena*** | **MNHN** **1888-6**  **Holotype** | **-** | **Edge of Tsijobonina, Menabé** |
| X | X | ***Hypogeomys antimena*** | **MNHN 1888-10 Paratype** | **M?** | **West Coast, edge Tsidibm and Andranounere, Menabé-** |
| X | X | ***Hypogeomys antimena*** | **MNHN 1888-8 Paratype** | **M?** | **West Coast, edge Tsidibm and Andranounere, Menabé** |
| X | - | ***Hypogeomys antimena*** | **MNHN 1888-7 Paratype** | **M?** | **West Coast, edge Tsidibm and Andranounere, Menabé** |
| X | X | ***Hypogeomys antimena*** | **MNHN 1888-9 Paratype** | **Juv** | **West Coast, edge Tsidibm and Andranounere, Menabé** |
| X | X | *Macrotarsomys bastardi bastardi* | BMNH 96.274 | M | Unknown locality |
| X | X | *Macrotarsomys bastardi bastardi* | BMNH 96.275 | - | Unknown locality |
| - | X | *Macrotarsomys bastardi bastardi* | BMNH 47.1626 | M | Ihosy, 35 miles N of |
| X | - | *Macrotarsomys bastardi bastardi* | BMNH 47.1636 | M | Ihosy, 35 miles N of |
| X | - | *Macrotarsomys bastardi bastardi* | BMNH 47.1633 | M | Ihosy, 35 miles N of |
| - | X | *Macrotarsomys bastardi bastardi* | BMNH 47.1632 | M | Ihosy, 35 miles N of |
| - | X | *Macrotarsomys bastardi bastardi* | BMNH 47.1646 | F | Ihosy, 35 miles N of |
| X | X | *Macrotarsomys bastardi bastardi* | BMNH 47.1643 | F | Ihosy, 35 miles N of |
| X | X | *Macrotarsomys bastardi bastardi* | FMNH 194627 | F | Toliara Province, Ankatrakatraka Forest |
| X | X | *Macrotarsomys bastardi bastardi* | FMNH 194662 | F | Toliara Province, south of Ankazoabo-Sud |
| X | X | *Macrotarsomys bastardi bastardi* | FMNH 176118 | M | Toliara Province, Kirindy Mité |
| X | X | *Macrotarsomys bastardi bastardi* | UADBA 19113 | - | Toliara Province, Tsimanampesotse |
| X | X | *Macrotarsomys bastardi bastardi* | UADBA 19122 | - | Toliara Province, Tsimanampesotse |
| **-** | **X** | ***Macrotarsomys bastardi bastardi*** | **MNHN 1912-134**  **Holotype** | **-** | **Lamboharana** |
| - | X | *Macrotarsomys bastardi bastardi* | MNHN 1957-787 | M | Lamboromakaudra, Sakaraha Forest |
| X | X | *Macrotarsomys bastardi bastardi* | MNHN 1992-1268 | - | Sihanamoro |
| X | - | *Macrotarsomys bastardi bastardi* | MNHN 1980-291 | - | West of Morondava |
| **X** | **X** | ***Macrotarsomys bastardi occidentalis*** | BMNH **47.1677** | **F** | **Beroboka, 40 miles N of Morondava** |
| X | X | *Macrotarsomys bastardi occidentalis* | BMNH 47.1703 | F | Tsihombe |
| X | X | *Macrotarsomys bastardi occidentalis* | BMNH 47.1675 | M | Beroboka, 40 miles N of Morondava |
| X | X | *Macrotarsomys bastardi occidentalis* | BMNH 47.1680 | F | Beroboka, 40 miles N of Morondava |
| X | X | *Macrotarsomys bastardi occidentalis* | BMNH 47.1688 | M | Tulear, 26 miles E of |
| - | X | *Macrotarsomys bastardi occidentalis* | BMNH 47.1699 | M | Tsihombe |
| X | - | *Macrotarsomys bastardi occidentalis* | BMNH 47.1698 | F | Tulear, 26 miles E of |
| X | - | *Macrotarsomys bastardi occidentalis* | FMNH 151955 | F | Toliara Province, Zombitsy Forest |
| X | X | *Macrotarsomys bastardi* | MNHN 1961-231 | M | Beroboka |
| X | X | *Macrotarsomys bastardi* | MNHN 1961-223 | M | Beroboka |
| X | X | *Monticolomys koopmani* | FMNH 165713 | M | Fianarantsoa Province, Andringitra |
| - | X | *Monticolomys koopmani* | FMNH 165714 | M | Fianarantsoa Province, Andringitra |
| X | X | *Monticolomys koopmani* | FMNH 165715 | F | Fianarantsoa Province, Andringitra |
| X | - | *Monticolomys koopmani* | FMNH 165716 | M | Fianarantsoa Province, Andringitra |
| - | X | *Monticolomys koopmani* | FMNH 165718 | F | Fianarantsoa Province, Andringitra |
| - | X | *Monticolomys koopmani* | FMNH 165717 | F | Fianarantsoa Province, Andringitra |
| X | X | *Monticolomys koopmani* | FMNH 165719 | F | Fianarantsoa Province, Andringitra |
| X | X | *Monticolomys koopmani* | FMNH 165720 | F | Fianarantsoa Province, Andringitra |
| X | X | *Monticolomys koopmani* | FMNH 165798 | F | Fianarantsoa Province, Andringitra |
| X | X | *Monticolomys koopmani* | FMNH 165800 | M | Fianarantsoa Province, Andringitra |
| - | X | *Monticolomys koopmani* | FMNH 166261 | F | Fianarantsoa Province, Andringitra |
| X | X | *Monticolomys koopmani* | FMNH 168039 | F | Fianarantsoa Province, Andringitra |
| X | X | *Monticolomys koopmani* | FMNH 168040 | F | Fianarantsoa Province, Andringitra |
| - | X | *Monticolomys koopmani* | FMNH 168041 | M | Fianarantsoa Province, Andringitra |
| X | - | *Monticolomys koopmani* | FMNH 165799 | F | Fianarantsoa Province, Andringitra |
| - | X | *Monticolomys koopmani* | FMNH 168044 | F | Fianarantsoa Province, Andringitra |
| - | X | *Monticolomys koopmani* | FMNH 151899 | F | Fianarantsoa Province, Andringitra |
| - | X | *Monticolomys koopmani* | FMNH 159508 | F | Fianarantsoa Province, Andringitra |
| - | X | *Monticolomys koopmani* | FMNH 161696 | M | Fianarantsoa Province, Andringitra |
| - | X | *Monticolomys koopmani* | FMNH 161697 | F | Fianarantsoa Province, Andringitra |
| - | X | *Monticolomys koopmani* | FMNH 161699 | M | Fianarantsoa Province, Andringitra |
| X | X | *Monticolomys koopmani* | FMNH 161700 | M | Fianarantsoa Province, Andringitra |
| X | X | *Monticolomys koopmani* | FMNH 162099 | M | Fianarantsoa Province, Ivohibe |
| - | X | *Monticolomys koopmani* | FMNH 168043 | M | Fianarantsoa Province, Andringitra |
| X | - | *Monticolomys koopmani* | FMNH 185767 | M | Fianarantsoa Province, Andringitra |
| - | X | *Monticolomys koopmani* | FMNH 185765 | F | Fianarantsoa Province, Andringitra |
| X | X | *Monticolomys koopmani* | UADBA 11805 | F | Fianarantsoa Province, Fandriana-Marolambo |
| X | X | *Nesomys audeberti* | FMNH 151696 | M | Fianarantsoa Province, Andringitra |
| X | X | *Nesomys audeberti* | FMNH 167568 | - | Fianarantsoa Province, Vevembe Forest |
| X | X | *Nesomys audeberti* | FMNH 169185 | M | Fianarantsoa Province, Ivohibe |
| X | X | *Nesomys audeberti* | FMNH 169186 | F | Fianarantsoa Province, Ivongo Forest |
| X | X | *Nesomys audeberti* | FMNH 169187 | F | Fianarantsoa Province, Ivongo Forest |
| X | X | *Nesomys audeberti* | FMNH 170845 | F | Fianarantsoa Province, Ranomafana |
| X | X | *Nesomys audeberti* | FMNH 170846 | M | Fianarantsoa Province, Ranomafana |
| X | X | *Nesomys audeberti* | FMNH 170847 | M | Fianarantsoa Province, Ranomafana |
| X | X | *Nesomys audeberti* | FMNH 170848 | M | Fianarantsoa Province, Ranomafana |
| X | X | *Nesomys audeberti* | UADBA 32553 | F | Toamasina Province, Torotorofotsy |
| X | X | *Nesomys audeberti* | UADBA 32643 | M | Toamasina Province, Analamay |
| X | - | *Nesomys audeberti* | MNHN 1962-2371 | F | 20 km west of Vondrozo |
| X | X | *Nesomys rufus* | FMNH 154058 | M | Mahajanga Province, Anjanaharibe-Sud |
| X | X | *Nesomys rufus* | FMNH 154059 | M | Mahajanga Province, Anjanaharibe-Sud |
| X | X | *Nesomys rufus* | FMNH 154061 | F | Mahajanga Province, Anjanaharibe-Sud |
| X | X | *Nesomys rufus* | FMNH 154062 | M | Mahajanga Province, Anjanaharibe-Sud |
| - | X | *Nesomys rufus* | FMNH 167648 | F | Fianarantsoa Province, Manambolo Forest |
| X | X | *Nesomys rufus* | FMNH 154063 | F | Mahajanga Province, Anjanaharibe-Sud |
| X | X | *Nesomys rufus* | FMNH 159475 | F | Antananarivo Province, Anjozorobe |
| X | X | *Nesomys rufus* | FMNH 159476 | F | Antananarivo Province, Anjozorobe |
| X | X | *Nesomys rufus* | FMNH 159477 | M | Antananarivo Province, Anjozorobe |
| X | X | *Nesomys rufus* | FMNH 166166 | M | Antananarivo Province, Anjozorobe |
| X | X | *Nesomys rufus* | FMNH 188676 | M | Antananarivo Province, Anjozorobe |
| X | X | *Nesomys rufus* | FMNH 188677 | F | Antananarivo Province, Anjozorobe |
| X | X | *Nesomys rufus* | FMNH 188756 | M | Antananarivo Province, Anjozorobe |
| X | X | *Nesomys rufus* | FMNH 172620 | F | Antsiranana Province, Marojejy |
| X | X | *Nesomys rufus* | FMNH 172619 | F | Antsiranana Province, Marojejy |
| X | X | *Nesomys rufus* | FMNH 170856 | F | Fianarantsoa Province, Andrambovato |
| X | X | *Nesomys rufus* | FMNH 170857 | M | Fianarantsoa Province, Andrambovato |
| X | X | *Nesomys rufus* | FMNH 170849 | F | Fianarantsoa Province, Ranomafana |
| X | X | *Nesomys rufus* | FMNH 170850 | F | Fianarantsoa Province, Ranomafana |
| X | X | *Nesomys rufus* | FMNH 170851 | M | Fianarantsoa Province, Ranomafana |
| - | X | *Nesomys rufus* | FMNH 170855 | F | Fianarantsoa Province, Andrambovato |
| X | X | *Nesomys rufus* | FMNH 167582 | F | Fianarantsoa Province, Manambolo Forest |
| X | X | *Nesomys rufus* | FMNH 167647 | M | Fianarantsoa Province, Manambolo Forest |
| X | X | *Nesomys rufus* | FMNH 167649 | F | Fianarantsoa Province, Manambolo Forest |
| X | X | *Nesomys rufus* | FMNH 167650 | F | Fianarantsoa Province, Manambolo Forest |
| - | X | *Nesomys rufus* | FMNH 161915 | M | Fianarantsoa Province, Ivohibe |
| X | X | *Nesomys rufus* | FMNH 161916 | F | Fianarantsoa Province, Ivohibe |
| X | X | *Nesomys rufus* | FMNH 162101 | F | Fianarantsoa Province, Ivohibe |
| X | X | *Nesomys rufus* | FMNH 162102 | F | Fianarantsoa Province, Ivohibe |
| X | X | *Nesomys rufus* | UADBA 31705 | - | Toamasina Province, Sahambaky |
| X | X | *Nesomys rufus* | UADBA 32517 | - | Fianarantsoa Province, Andrambovato |
| X | - | *Nesomys rufus* | BMNH 35.1.8.327 | F | 20 km west of Vondrozo |
| - | X | *Nesomys rufus* | BMNH 35.1.8.326 | M | 20 km west of Vondrozo |
| X | X | *Nesomys rufus* | BMNH 97.9.1.159 | M | Ampitambe |
| X | X | *Nesomys rufus* | BMNH 35.1.8.335 | F | Andraga |
| X | X | *Voalavo gymnocaudus* | UADBA 10847 | - | Antsiranana Province, Marojejy |
| X | X | *Voalavo gymnocaudus* | FMNH 156162 | - | Antsiranana Province, Anjanaharibe-Sud |
| - | X | *Voalavo gymnocaudus* | FMNH 159648 | - | Mahajanga Province, Anjanaharibe-Sud |
| X | X | *Voalavo gymnocaudus* | FMNH 159725 | M | Antsiranana Province, Marojejy |
| X | X | *Voalavo gymnocaudus* | FMNH 159727 | F | Antsiranana Province, Marojejy |
| X | X | *Voalavo gymnocaudus* | FMNH 167476 | M | Mahajanga Province, Anjanaharibe-Sud |
| X | X | *Voalavo gymnocaudus* | FMNH 167473 | M | Mahajanga Province, Anjanaharibe-Sud |
| X | X | *Voalavo gymnocaudus* | FMNH 167474 | F | Mahajanga Province, Anjanaharibe-Sud |
| X | X | *Voalavo gymnocaudus* | FMNH 167475 | F | Mahajanga Province, Anjanaharibe-Sud |
| X | X | *Voalavo gymnocaudus* | FMNH 154041 | F | Mahajanga Province, Anjanaharibe-Sud |
| **X** | **X** | ***Voalavo gymnocaudus*** | **FMNH 154040**  **Holotype** | **M** | Antsiranana Province, Anjanaharibe-Sud |
