# Supplementary material for: Skull morphological evolution in Malagasy endemic Nesomyinae rodents
Source: PLoS One. 2022 Feb 4;17(2):e0263045. doi: 10.1371/journal.pone.0263045 (PMC8815910; doi:10.1371/journal.pone.0263045)
Supplement: S2 Table — Descriptions and types of the 27 landmarks used for the dorsal view. (DOCX) [file pone.0263045.s006.docx]

**Supplementary Table 2 Nesomyinae rodent geometrics morphometrics**

**Terray et al.**

**Table S2.** Descriptions and types of the 27 landmarks used for the dorsal view.

| **Landmark** | **Description** | **Type** |
| --- | --- | --- |
| **1** | Point at the anterior margin of the rostrum on suture | **I** |
| **2** | Left anterior point between nasal and premaxillary on suture | **I** |
| **3** | Point at the right anterior between nasal and premaxillary on suture | **I** |
| **4** | Point at the anterior end of the left infraorbital process | **II** |
| **5** | Point at the anterior end of the right infraorbital process | **II** |
| **6** | Point at posterior extremity of the left orbit | **II** |
| **7** | Point at posterior extremity of the right orbit | **II** |
| **8** | Left junction point between premaxillary, frontal and lacrimal | **I** |
| **9** | Right junction point between premaxillary, frontal and lacrimal | **I** |
| **10** | Left junction point between premaxillary, frontal and nasal | **I** |
| **11** | Right junction point between premaxillary, frontal and nasal | **I** |
| **12** | Point at the connection between the nasal-frontal suture and frontal suture | **I** |
| **13** | Point at the anterior end of the left temporal fenestra | **II** |
| **14** | Point at the anterior end of the right temporal fenestra | **II** |
| **15** | Left most outermost point of maxillo-frontal suture | **I** |
| **16** | Right most outermost point of maxillo-frontal suture | **I** |
| **17** | Left most outermost point of the front-temporal suture | **I** |
| **18** | Right most outermost point of the front-temporal suture | **I** |
| **19** | Left junction point between frontal, temporal and parietal | **I** |
| **20** | Right junction point between frontal, temporal and parietal | **I** |
| **21** | Point at posterior end of left temporal fenestra | **II** |
| **22** | Point at posterior end of right temporal fenestra | **II** |
| **23** | Junction between frontal-parietal suture and sagittal suture | **I** |
| **24** | Junction point between sagittal suture and interparietal-parietal suture | **I** |
| **25** | Left junction between the parietal-interparietal suture and the interparietal-occipital suture | **I** |
| **26** | Right junction point between the parietal-interparietal suture and the interparietal-occipital suture | **I** |
| **27** | Point on interparieto-occipital suture at level of the occipital protuberance | **II** |
