# Supplementary material for: Skull morphological evolution in Malagasy endemic Nesomyinae rodents
Source: PLoS One. 2022 Feb 4;17(2):e0263045. doi: 10.1371/journal.pone.0263045 (PMC8815910; doi:10.1371/journal.pone.0263045)
Supplement: S3 Table — Descriptions and types of the 42 landmarks used for the ventral view. (DOCX) [file pone.0263045.s007.docx]

**Supplementary Table 3 Nesomyinae rodent geometrics morphometrics**

**Terray et al.**

**Table S3.** Descriptions and types of the 42 landmarks used for the ventral view.

| **Landmark** | **Description** | **Type** |
| --- | --- | --- |
| **1** | Point at the anterior margin of the nasal on the suture | **I** |
| **2** | Most buccal point in the insertion of the right incisor | **I** |
| **3** | Most buccal point in the insertion of the left incisor | **I** |
| **4** | Earliest point of the right incisive foramen | **II** |
| **5** | Earliest point of the left incisive foramen | **II** |
| **6** | Posterior point of the right incisive foramen | **II** |
| **7** | Posterior point of the left incisive foramen | **II** |
| **8** | Most mesial point of the M1 plateau right side | **II** |
| **9** | Most mesial point of the M1 plateau left side | **II** |
| **10** | Buccal point at the widest point of the M1 right side | **II** |
| **11** | Lingual point at the widest point of the right M1 | **II** |
| **12** | Lingual point at the widest point of the left M1 | **II** |
| **13** | Mesial point at the widest point of the left M1 | **II** |
| **14** | Buccal point at the junction between the M1 and M2 right side | **III** |
| **15** | Lingual point at the junction between the M1 and M2 right side | **III** |
| **16** | Lingual point at the junction between the M1 and M2 left side | **III** |
| **17** | Buccal point at the junction between the M1 and M2 left side | **III** |
| **18** | Buccal point at the junction between the M2 and M3 right side | **III** |
| **19** | Lingual point at the junction between the M2 and M3 right side | **III** |
| **20** | Lingual point at the junction between the M2 and M3 left side | **III** |
| **21** | Buccal point at the junction between the M2 and M3 left side | **III** |
| **22** | Most distal point of the right M3 | **II** |
| **23** | Most distal point of the left M3 | **II** |
| **24** | Transition point between vomer and palatine on central suture | **I** |
| **25** | Central point of the suture between the presphenoid and the basiphenoid | **I** |
| **26** | Point at anterior end of the right basisphenoid slit | **II** |
| **27** | Point at posterior end of left basiphenoid slit | **II** |
| **28** | Posterior point of the right squamosal foramen | **II** |
| **29** | Posterior point of the left squamosal foramen | **II** |
| **30** | Anterior point of the right tympanic bulla | **III** |
| **31** | Anterior point of the left tympanic bulla | **III** |
| **32** | Internal point at the maximum width of the right tympanic bulla | **III** |
| **33** | Internal point at the maximum width of the left tympanic bulla | **III** |
| **34** | Exterior point at the maximum width of the right tympanic bulla | **III** |
| **35** | External point at the maximum width of the left tympanic bulla | **III** |
| **36** | Point on the right junction between basioccipital and basispheniod | **I** |
| **37** | Point on the left junction between basioccipital and basisphenoid | **I** |
| **38** | Posterior point of the right tympanic bulla | **III** |
| **39** | Posterior point of the left tympanic bulla | **III** |
| **40** | Anteroventral point of the foramen magnum | **II** |
| **41** | Right-most lateral point of the foramen magnum | **III** |
| **42** | Left-most lateral point of the foramen magnum | **III** |
